# Machine learning model for age related macular degeneration based on pesticides: the National Health and Nutrition Examination Survey 2007–2008

**DOI:** 10.3389/fpubh.2025.1561913

**Published:** 2025-04-16

**Authors:** Jiankang Liu, Bingli Wang, Qiuming Li

**Affiliations:** The First Affiliated Hospital of Zhengzhou University, Zhengzhou, China

**Keywords:** age related macular degeneration, pesticides, machine learning, NHANES, cross-section study

## Abstract

Age-related macular degeneration (AMD) is the most common cause of irreversible deterioration of vision in older adults. Previous studies have found that exposure to pesticides can lead to a worsening of AMD. In this paper, information on pesticide exposure and AMD from the National Health and Nutrition Examination Survey (NHANES) database was used to divide the data into a training set and a validation set. Firstly, the correlation between the variables in the model is analyzed. The model is then built using nine machine learning algorithms and verified on a validation set. Finally, it is found that the random forest model has high predictive value, and its Receiver Operating Characteristic (ROC) value is 0.75. Finally, SHapley additive interpretation (SHAP) analysis was used to rank the importance of each variable in the random forest model, and it was found that chlorpyrifos and malathion had quite significant effects on the occurrence and development of AMD.

## Introduction

Age-related macular degeneration (AMD) affects one in eight people over the age of 60 in developed countries and is the most common cause of irreversible blindness in older adults in developed countries. According to a comprehensive estimate, there are approximately 200 million people worldwide with AMD, and this number is expected to rise to nearly 300 million by 2040 (1). In the United States, geographic atrophy (geographic atrophy) of AMD accounts for one-fifth of the legal standard of blindness (2). Early AMD primarily manifests as clinical symptoms such as drusen and changes in the retinal pigment epithelium. Clinically, late-stage AMD is mainly divided into neovascular (also known as wet or exudative) AMD and non-neovascular (also known as atrophic, dry or non-exudative) AMD. As AMD progresses to the late stage, it leads to vision loss in the macula, which is irreversible and ultimately leads to complete loss of vision (3). Based on current research findings, both genetic factors and environmental factors play a role in the pathogenesis of AMD (4). AMD is associated with polymorphisms in about 20 genes (5, 6). Smoking is known to be associated with an increased risk of AMD, and obesity may also be an important factor (7). However, in addition to these, there are other pathogenic factors that also play a role (8).

Pesticides, as a modern industrial chemical product, are widely used for various purposes, including pest control in agriculture and pesticides in daily living environments. According to the United Nations Food and Agriculture Organization (FAO), global pesticide use exceeded 2.5 million tons in 2020 (9). The use of pesticides to some extent improves the quantity and quality of agricultural products and improves people’s living environment, but its indiscriminate and irrational use also has a huge impact on the environment and human beings themselves (10). For example, after the use of pesticides, most of them cannot be degraded in a short time, resulting in residues in food, soil, and the environment. Due to the amplification effect of the food chain and the biological magnification, these residues will ultimately affect human beings themselves (11).

Previous studies have focused on the toxicology and treatment of pesticide acute and chronic poisoning, while there has been less research on the impact of chronic pesticide exposure on human tissues. Fareed et al. reported cases of pesticide workers who were more likely to develop AMD (12). There has been even less research on the relationship between chronic pesticide exposure and retinal degeneration. Only Martha et al. studied found that greater pesticide exposure was more likely to lead to the development of AMD (8). These studies often limit themselves to estimating the relationship between single pesticide exposure and AMD, ignoring the synergistic effects of different types of pesticides on the development of AMD, and are often limited to small sample studies, with conclusions that have certain limitations.

NHANES is a nationwide representative survey conducted by the National Center for Health Statistics (NCHS) of the US. The study uses a complex multi-stage stratified and probability sampling method to assign different weights to participants and uses a series of questionnaires and laboratory tests to assess the health and nutritional status of non-institutionalized US civilians. NHANES is conducted every 2 years, and all survey data can be accessed from the nhanes.cdc.gov website. This survey has been approved by the National Health Statistics Ethics Review Board. Written informed consent has been obtained from all participants according to the Helsinki Declaration (13).

Machine learning methods such as decision trees, random forests, and neural networks are a way to build models that can analyze complex nonlinear relationships, allowing for a more accurate representation of the real-world relationship between pesticide exposure and AMD risk. Furthermore, machine learning algorithms have the ability to select features, enabling them to automatically identify and select important variables. For example, random forests and gradient boosted trees provide importance scores. Therefore, this study uses machine learning algorithms to analyze the pesticide exposure and AMD data from the NHANES database for the US population, analyzes the correlation between the two and builds a model to evaluate the model’s value, thereby discovering the relationship between pesticide exposure and AMD.

## Methods

**Figure 1:**
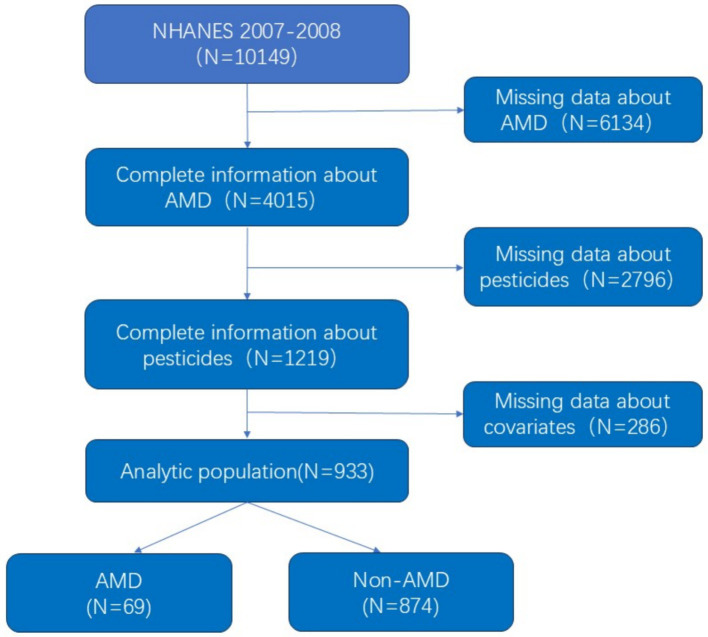
Study participants included for the present analysis from the 2007 to 2008 NHANES.

The study used data from the NHANES 2007– 2008 survey, which included a total of 10,149 participants. Retinal photographs were taken for participants aged 40 or older, and the images were analyzed to determine if the participants had age-related macular degeneration. A total of 6,134 participants who did not have retinal images were excluded, as were 2,796 participants who did not have information on pesticide exposure. In addition, participants without income, smoking, drinking, hypertension, etc. were not included in the study, and a total of 933 participants were included in the study, as shown in Figure 1.

### Definition of variables

Definition of Age-Related Macular Degeneration (AMD): AMD information is determined through retinal photography images, captured using the Canon Non-Mydriatic Retinal Camera CR 6–45 NM from participants aged 40 and older. Digital images of the retina are captured at a 45-degree angle without dilation using the Canon Non-Mydriatic Retinal Camera CR 6–45 NM. Technicians who perform the examinations have received training in using digital imaging systems. Digital images are evaluated by graders at the University of Wisconsin to determine the grade. The retinal images are divided into three severity categories: none, early, and late. In order to further investigate the relationship between AMD and pesticide exposure, in this study, both “early and late AMD” are considered to have AMD.

Acquisition of Pesticide Exposure Data: Pesticide exposure data is determined using urine testing data collected during MEC check-up vehicles. The target analytes are extracted and concentrated from the urine matrix using an automated solid phase extraction system. Selective separation of the analytes is achieved using high-performance liquid chromatography with a gradient elution program. Sensitive detection of the analytes is performed by a triple quadrupole mass spectrometer with a heated electrospray ionization source. Analytes are identified using the specific m/z ion transition, the retention time and the ion ratio of the quantification and confirmation m/z ion transitions. Isotopically labeled internal standards are used for precise and accurate quantification. This method can be used to assess human exposure to select non-persistent pesticides by measuring their metabolites in urine. It does not directly test for any disease (14).

According to previous studies, some factors that may affect the occurrence of AMD were also included in this study, including age, gender, race, education level, marital status, income, and other demographic information, as well as smoking, drinking, and whether the person has hypertension (7). Age and family income in demographic information were included as continuous variables, while race, marital status, gender and education level were included as categorical variables. In addition, information on smoking, alcohol consumption, and hypertension was obtained through questionnaires and included as a binary variable in the model.

### Data analysis

All data analysis was conducted using R software. Continuous variables were expressed as means and standard errors (SE), and categorical variables were expressed as percentages and SE. Chi-square test or *t*-test was used to compare the baseline characteristics of participants. The data were divided into a training set and a validation set in a 3:1 ratio. The optimal model type was determined by using the training set data with a five-fold cross-validation method to infer the performance of each model in multiple training sessions and with certain evaluation criteria, focusing on the overall performance of each model. In the training set, we used nine different ML construction models, including neural network (enet), support vector machine (rsvm), LASSO regression (mlp), gradient boosting machine (lightgbm), logistic regression (logistic), XGBoost (xgboost), C5.0 decision trees (dt), K-nearest neighbor (knn), and random forest (rf), and validated the models in the validation set. We use the tidymodels package in R software to integrate various models, transfer the parameters of each model, use the function of the corresponding model to perform data analysis, set up five-fold cross-validation, use grid search to find the optimal hyperparameters, and build the model with the optimal hyperparameters found.

SHAP values were calculated for each feature in the random forest model using the shap package in R. This package implements the TreeSHAP algorithm, which is optimized for tree-based models like random forests. For each observation in the validation set, the SHAP values were computed to quantify the contribution of each feature to the predicted risk of AMD. A feature importance bar chart was generated by aggregating the absolute SHAP values across all observations. This chart ranks features based on their overall impact on the model’s predictions, helping to identify the most influential features. SHAP summary plots were created to visualize the distribution of SHAP values for each feature. These plots show how the value of a feature influences the model’s output, with each point representing an observation. Additionally, force plots were generated for individual predictions to illustrate how each feature contributes to shifting the model’s output from the base value (the average model output) to the final prediction. SHAP interaction values were calculated to explore the interaction effects between pairs of features. These values quantify how the combined effect of two features differs from their individual contributions. SHAP analysis, based on the Shapley value in cooperative game theory, provides a quantitative analysis of the contribution of features to the model output (15). On the one hand, SHAP analysis allows the calculation of the overall impact of each pesticide exposure variable on the prediction of AMD risk. This helps identify which pesticide is an important driver of AMD risk. On the other hand, SHAP analysis can also reveal the interaction effects between pesticide exposures and how they jointly affect AMD risk (16).

## Results

**Figure 2:**
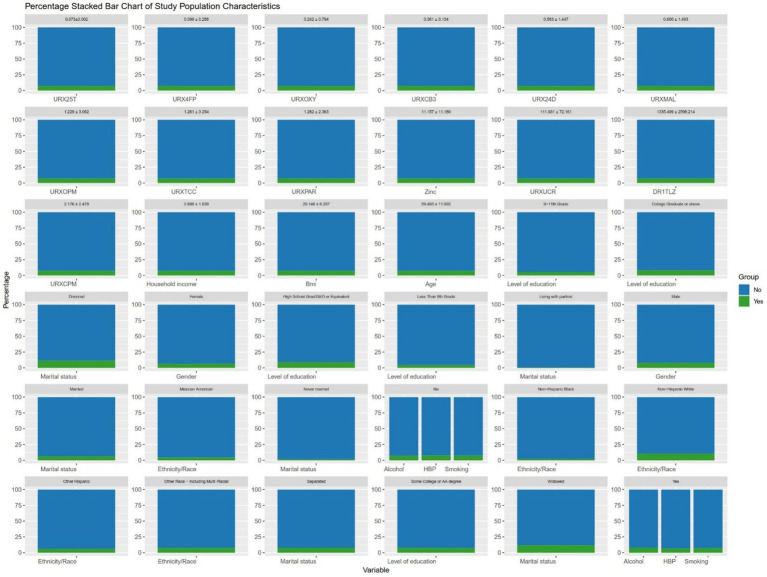
The distribution of the population included in the study. To make the chart more concise, abbreviations have been used for some variable names, as detailed in Appendix A.

**Figure 3:**
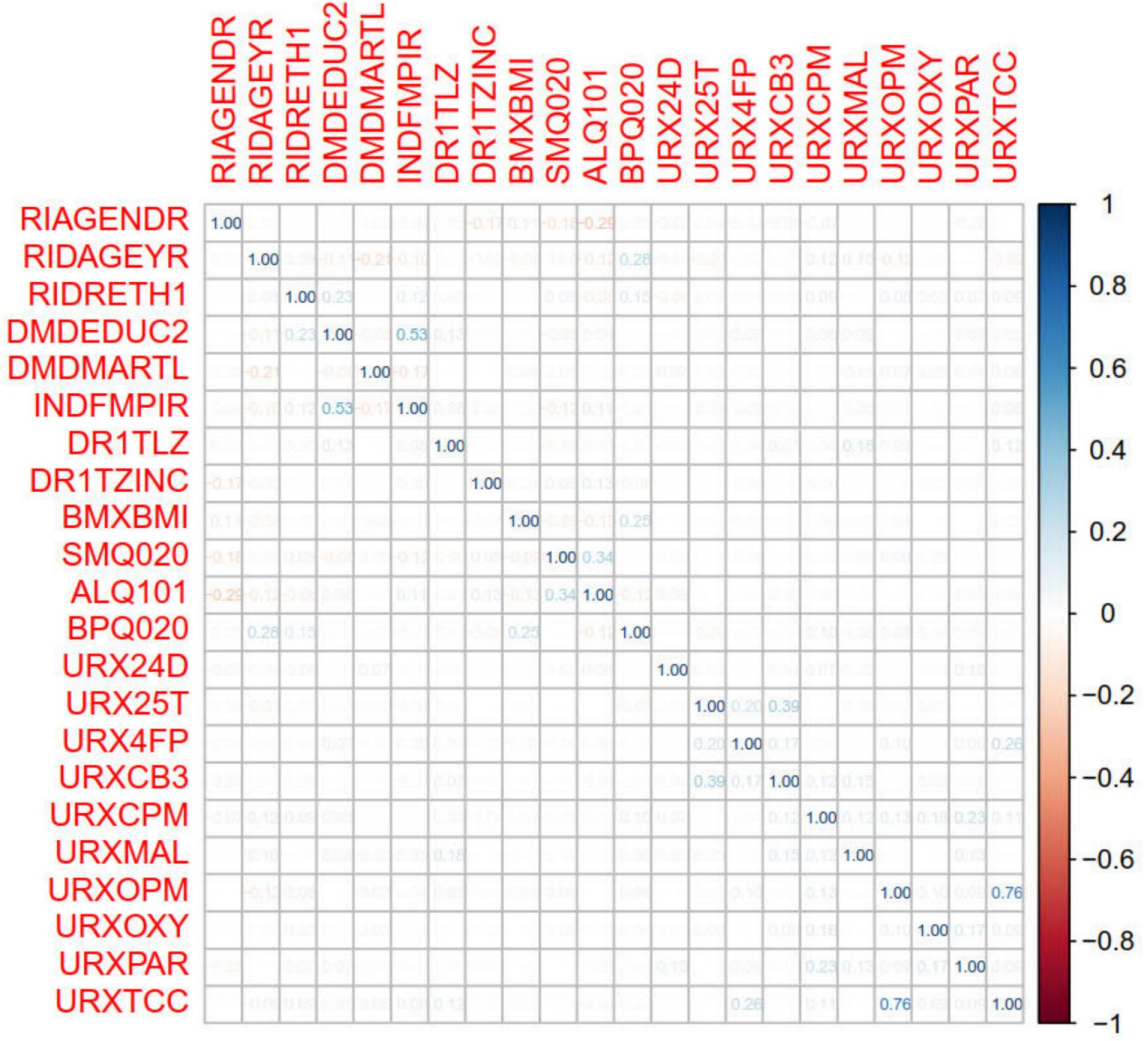
Results of Pearson correlation analysis among different variables. In order to make the chart more concise, some variables are replaced by their short words.

It can be seen in Table 1, similar to previous studies, the average age of participants with AMD was higher than that of participants without AMD (70.014 ± 11.710 vs. 59.494 ± 11.294). In addition, there were significant differences in race, marital status, and Malathion diacid content in urine between AMD patients and non-AMD patients (Figure 2). After that, we conducted Pearson correlation tests between the variables included in the model to check for any significant correlations, as shown in the Figure 3, except for a weak correlation between 3-phenoxybenzoic and dichlorovnl-dimeth prop carboacid, no other significant correlations were found.

**Table 1:** Description of baseline characteristics of the population included in the study.

	No (*N* = 874)	Yes (*N* = 69)	Standardize diff.	*p*-value
Age	59.495 ± 11.992	70.014 ± 11.710	0.888 (0.639, 1.136)	<0.001
Gender			0.089 (−0.157, 0.334)	0.479
Male	430 (49.199%)	37 (53.623%)		
Female	444 (50.801%)	32 (46.377%)		
Ethnicity/Race			0.551 (0.305, 0.797)	0.004
Mexican American	134 (15.332%)	6 (8.696%)		
Other Hispanic	92 (10.526%)	6 (8.696%)		
Non-Hispanic White	436 (49.886%)	50 (72.464%)		
Non-Hispanic Black	176 (20.137%)	4 (5.797%)		
Other Race—including multi-racial	36 (4.119%)	3 (4.348%)		
Level of education			0.266 (0.020, 0.511)	0.553
Less than 9th grade	132 (15.103%)	6 (8.696%)		
9–11th grade (includes 12th grade with no diploma)	138 (15.789%)	8 (11.594%)		
High school grad/GED or equivalent	211 (24.142%)	21 (30.435%)		
Some college or AA degree	205 (23.455%)	17 (24.638%)		
College graduate or above	188 (21.510%)	17 (24.638%)		
Marital status			0.512 (0.266, 0.758)	0.02
Married	509 (58.238%)	34 (49.275%)		
Widowed	112 (12.815%)	15 (21.739%)		
Divorced	126 (14.416%)	16 (23.188%)		
Separated	35 (4.005%)	3 (4.348%)		
Never married	60 (6.865%)	1 (1.449%)		
Living with partner	32 (3.661%)	0 (0.000%)		
Smoking			0.014 (−0.231, 0.259)	0.913
No	450 (51.487%)	36 (52.174%)		
Yes	424 (48.513%)	33 (47.826%)		
Alcohol use			0.067 (−0.179, 0.312)	0.591
No	585 (66.934%)	44 (63.768%)		
Yes	289 (33.066%)	25 (36.232%)		
Hypertension			0.076 (−0.169, 0.321)	0.541
No	410 (46.911%)	35 (50.725%)		
Yes	464 (53.089%)	34 (49.275%)		
Household income	2.698 ± 1.639	2.395 ± 1.579	0.188 (−0.057, 0.434)	0.138
Lutein and zeaxanthin	1335.499 ± 2598.214	2018.377 ± 5405.980	0.161 (−0.084, 0.406)	0.059
Zinc	11.157 ± 11.180	13.567 ± 18.147	0.160 (−0.085, 0.405)	0.103
BMI	29.146 ± 6.267	28.251 ± 5.227	0.155 (−0.090, 0.400)	0.248
URX24D	0.583 ± 1.447	0.773 ± 1.545	0.127 (−0.118, 0.373)	0.295
URX4FP	0.099 ± 0.288	0.115 ± 0.236	0.060 (−0.185, 0.305)	0.658
URXCB3	0.361 ± 0.134	0.350 ± 0.000	0.113 (−0.132, 0.358)	0.507
URXCPM	2.176 ± 2.479	2.533 ± 2.728	0.137 (−0.108, 0.382)	0.254
URXMAL	0.660 ± 1.493	1.136 ± 3.159	0.192 (−0.053, 0.438)	0.023
URXOPM	1.229 ± 3.062	0.905 ± 1.499	0.134 (−0.111, 0.380)	0.384
URXOXY	0.242 ± 0.794	0.298 ± 0.664	0.076 (−0.169, 0.321)	0.573
URXPAR	1.282 ± 2.363	1.087 ± 1.535	0.098 (−0.147, 0.343)	0.5
URXTCC	1.261 ± 3.254	1.243 ± 3.003	0.006 (−0.239, 0.251)	0.964
URXUCR	111.931 ± 72.161	104.000 ± 59.900	0.120 (−0.126, 0.365)	0.374
URX25T	0.073 ± 0.002	0.007 ± 0.000	0.083 (−0.162, 0.328)	0.971

**Figure 4:**
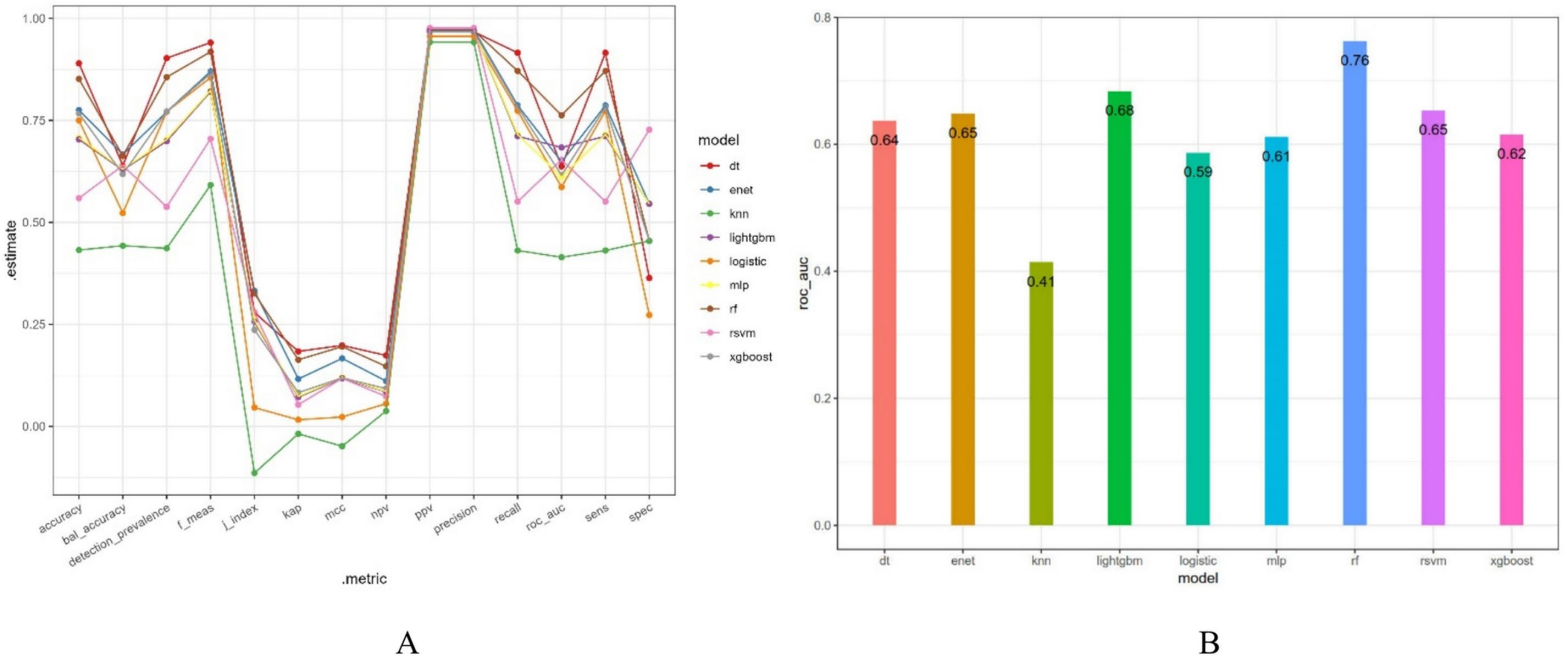
Various evaluation indicators of different models. bal_accuracy: Balanced Accuracy; f_meas: F1 Score; j_index: Youden’s J Index; kap: Cohen’s Kappa; mcc: Matthews Correlation Coefficient; npv: Negative Predictive Value; ppv: Positive Predictive Value; sens: Sensitivity; spec: Specificity.

**Figure 5:**
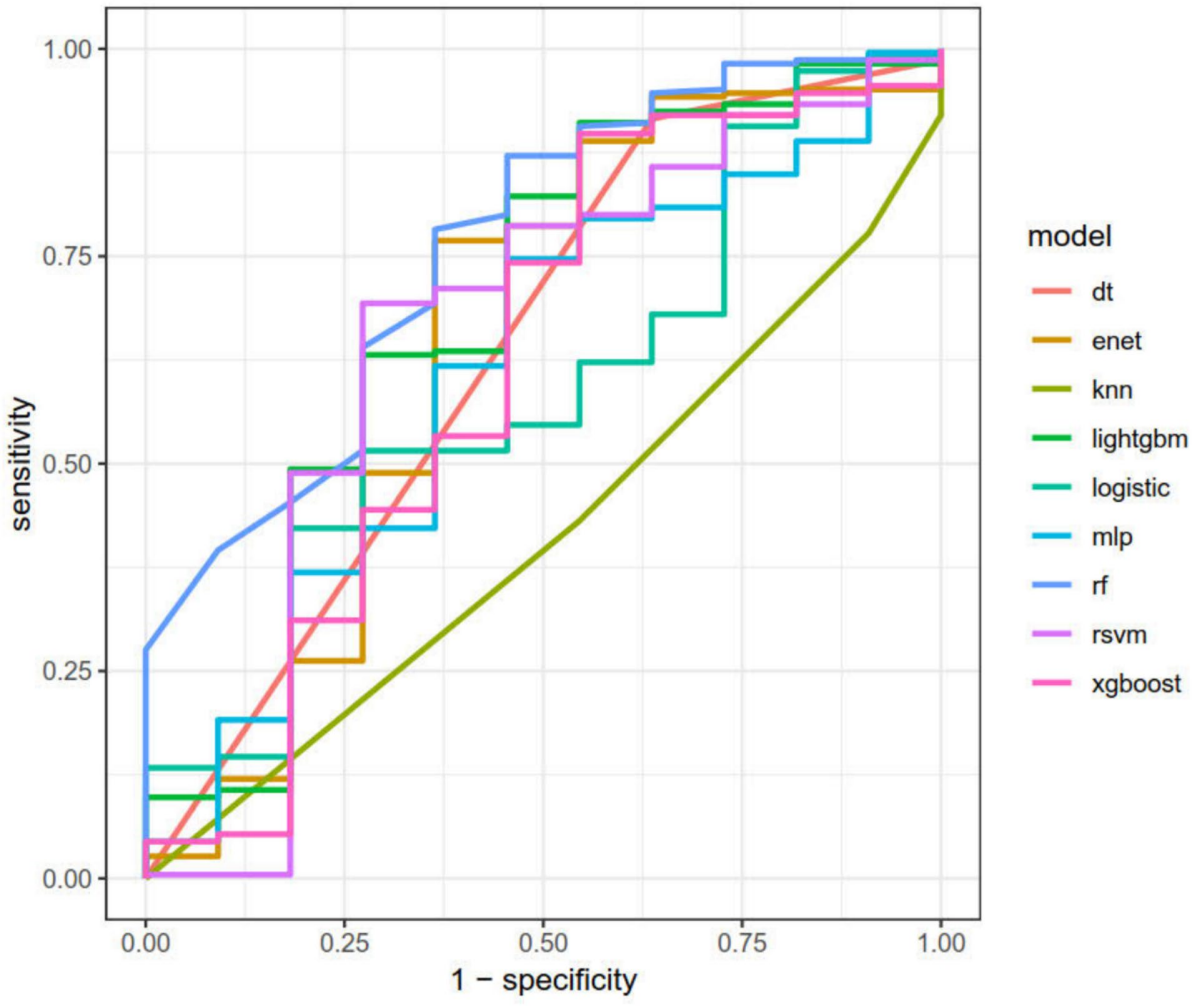
The area under the curve for the nine models. It can be seen from the figure that among all models, the random forest model has the highest predictive value on the validation set (AUC = 0.75).

**Figure 6:**
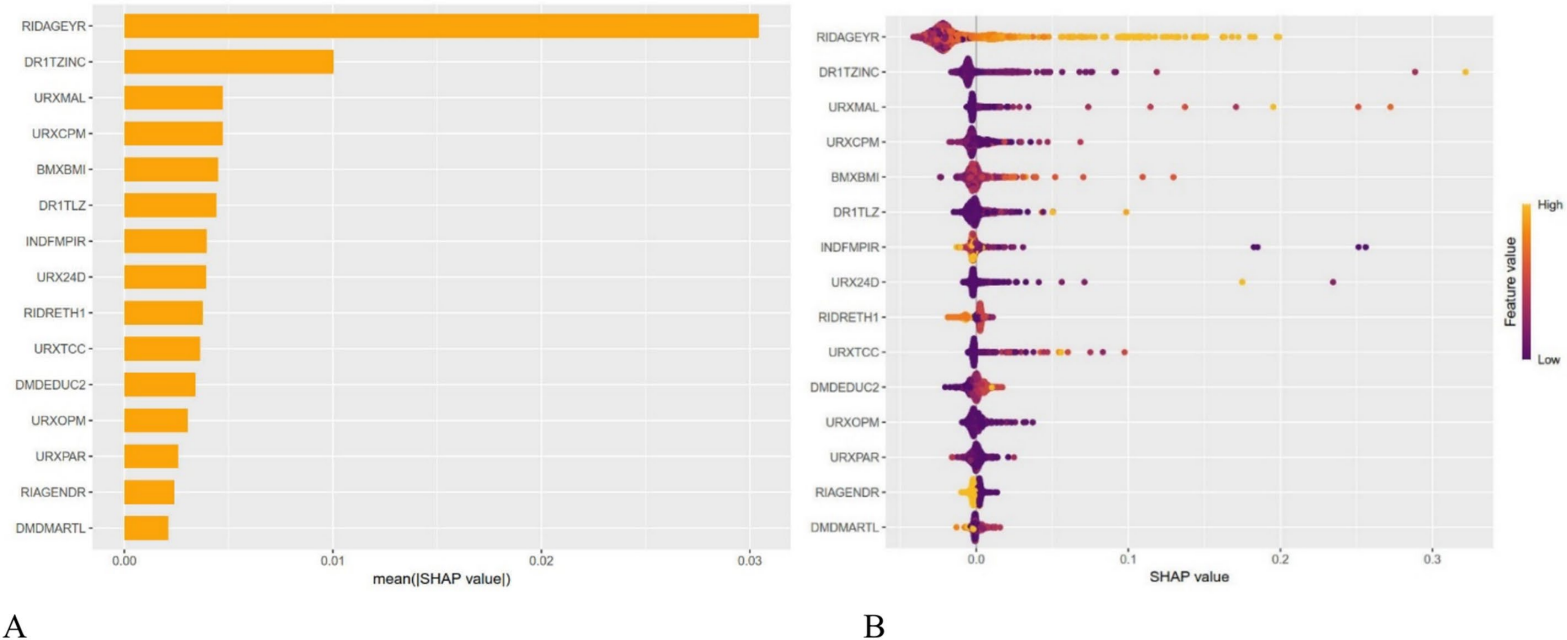
The shap values for different variables in the random forest model. It can be seen from the figure that the top three variables of importance are age, zinc intake, and Malathion in turn.

**Figure 7:**
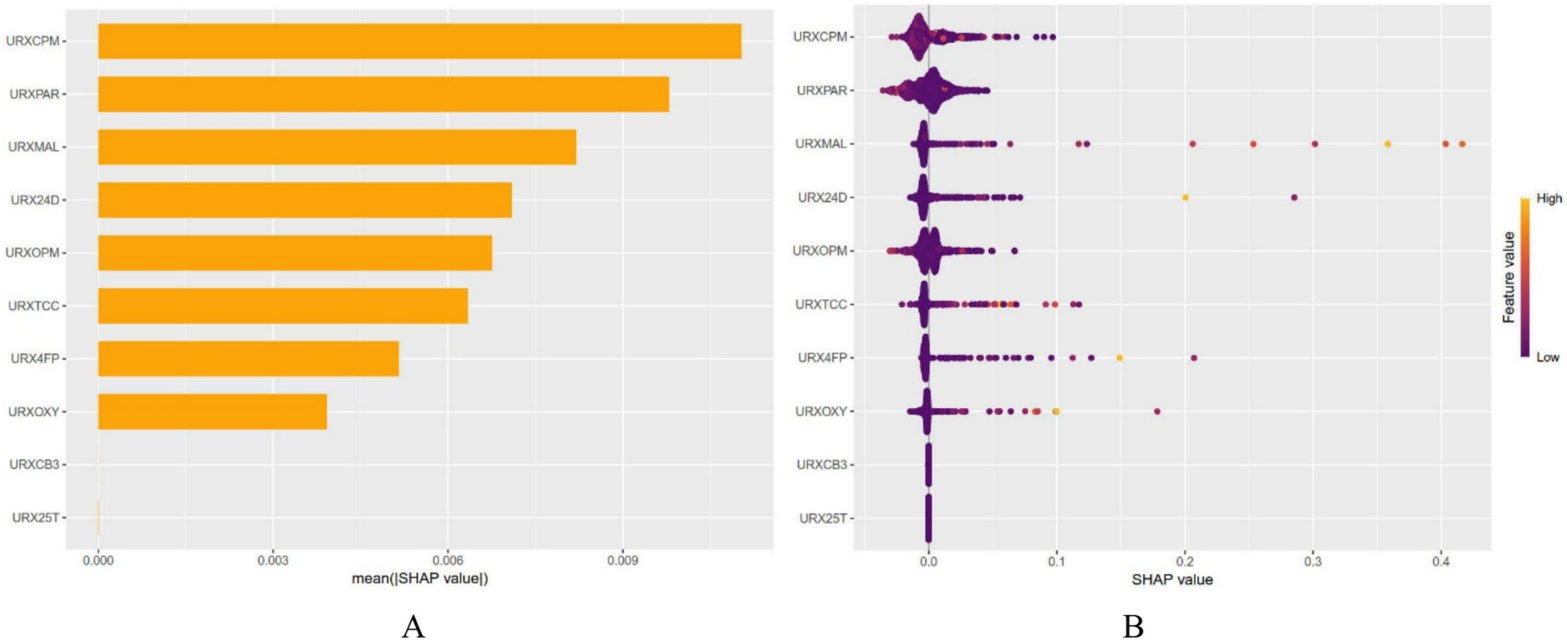
The shap values of each variable after only pesticide variables are included in the random forest model for importance ranking. The top three important variables are 3,5,6-trichloropyridinol, Paranitrophenol, and Malathion diacid.

**Figure 8:**
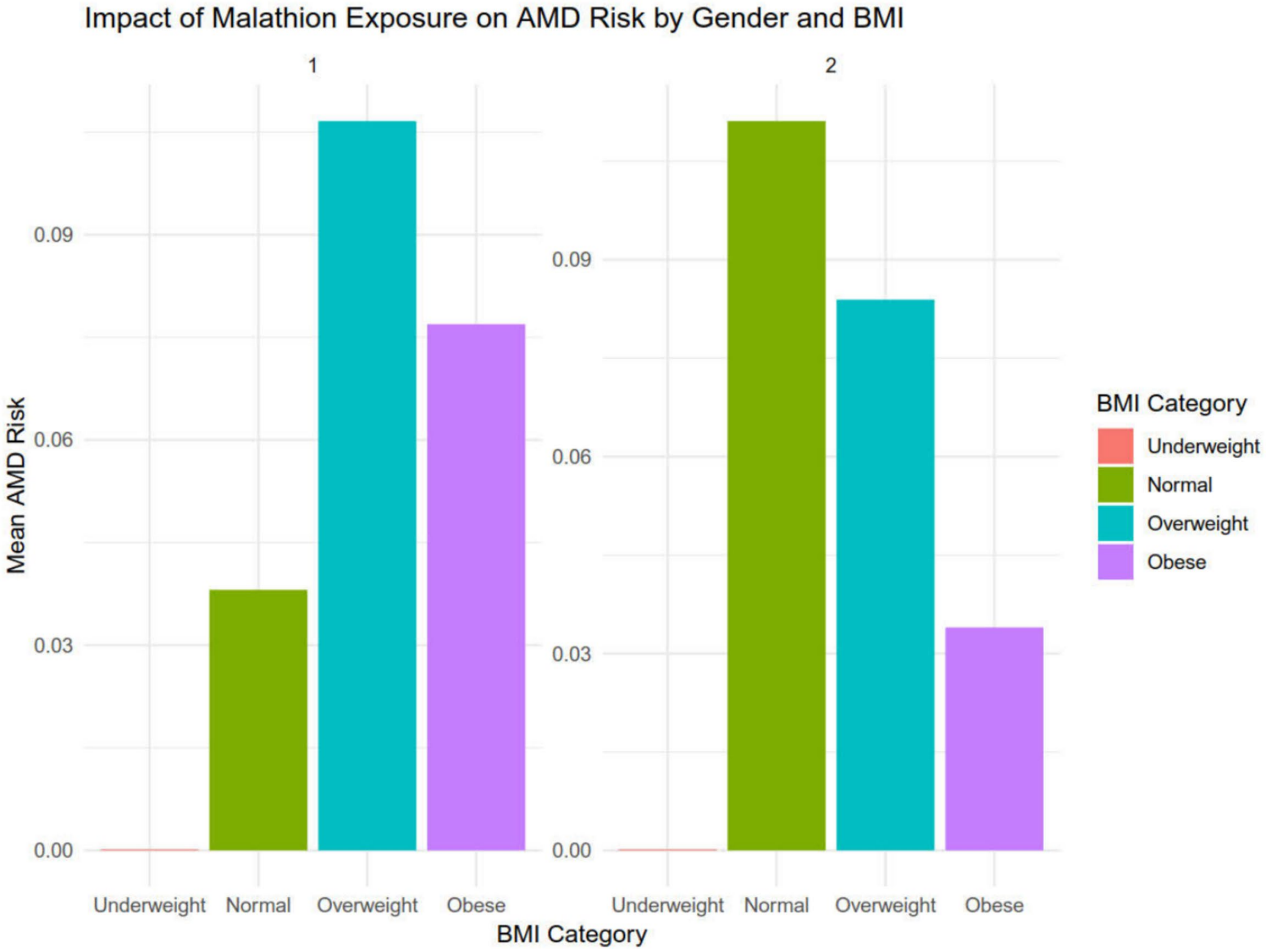
The effects of malathion exposure on the development of AMD in men and women with different BMI.

**Figure 9:**
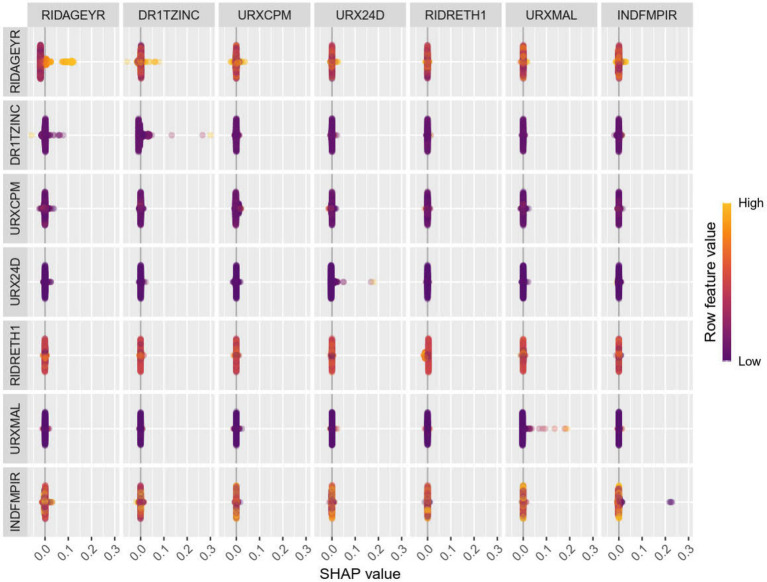
The shap correlation between the different variables.

After dividing the data into a training set and a validation set in a 3:1 ratio, the model was trained using the training set and validated on the validation set. The ROC curve for AMD prediction risk was fitted on the validation set, and the model parameters for each model are shown in Figure 4 and Table 2. It can be seen that among the nine models, the model using the random forest algorithm has the highest area under the curve (Figure 5), the optimal hyperparameters of each model are detailed in Appendix B. After conducting SHAP analysis on the random forest model, the feature importance bar chart was drawn, which shows the importance scores of each feature and helps quickly identify key features (Figures 6A,B). The feature importance bar chart shows that when demographic variables are included, the top three most important elements are age, zinc intake, and Malathion, while when only pesticide variables included (Figures 7A,B) the top three most important pesticide types are Chlorpyrifos, Paranitrophenol, and Malathion, indicating that Malathion is the most important exposure factor affecting AMD compared to other included pesticides. Figure 8 shows the effects of malathion exposure on the development of AMD in men and women with different BMIs. It can be seen that among male participants, those who are overweight have the highest risk of malathion exposure, while among female participants, those who are normal weight have the highest tendency to develop AMD. In addition, we conducted shap correlation analysis on the variables with top shap values in the random forest model, and the results are shown in Figure 9. It can be seen that age has the greatest interaction with zinc intake, while the other variables have less significant effects.

**Table 2:** The specific metrics of the nine models in the validation set.

model	accuracy	kap	sens	spec	ppv	npv	mcc	j_index	bal_accuracy	detection_prevalence	precision	recall	f1 score	roc_auc
logistic	0.75	0.0161	0.773	0.273	0.956	0.0556	0.0231	0.0461	0.523	0.771	0.956	0.773	0.855	0.587
enet	0.775	0.116	0.787	0.545	0.973	0.111	0.167	0.332	0.666	0.771	0.973	0.787	0.87	0.648
dt	0.89	0.184	0.916	0.364	0.967	0.174	0.198	0.279	0.64	0.903	0.967	0.916	0.941	0.637
rf	0.852	0.163	0.871	0.455	0.97	0.147	0.195	0.326	0.663	0.856	0.97	0.871	0.918	0.762
xgboost	0.767	0.0828	0.782	0.455	0.967	0.0926	0.119	0.237	0.618	0.771	0.967	0.782	0.865	0.615
rsvm	0.559	0.0532	0.551	0.727	0.976	0.0734	0.118	0.278	0.639	0.538	0.976	0.551	0.705	0.653
mlp	0.708	0.0735	0.716	0.545	0.97	0.0857	0.12	0.261	0.631	0.703	0.97	0.716	0.824	0.612
lightgbm	0.703	0.0714	0.711	0.545	0.97	0.0845	0.118	0.257	0.628	0.699	0.97	0.711	0.821	0.684
knn	0.432	−0.0182	0.431	0.455	0.942	0.0376	−0.0486	−0.114	0.443	0.436	0.942	0.431	0.591	0.415

## Discussion

As a human-made chemical agent used to kill pests and weeds in agriculture, pesticides can cause unavoidable effects on human tissues if consumed in excess or chronically exposed. For example, organophosphate pesticide poisoning, the most common pesticide poisoning, can be divided into acute and chronic poisoning, both of which can cause serious harm to human life safety. Studies have found that the accumulation of a variety of pesticides, including organophosphates, in the body can lead to the development of retinal.

This study used the data on heavy metal exposure from the 2007–2008 National Health and Nutrition Examination Survey (NHANES) of the United States and used nine machine learning algorithms to screen the data. An effective prediction model for predicting AMD risk based on pesticide exposure levels was finally developed. We not only revealed the best performance of the RF model through SHAP analysis, but also proved that, besides age factor, urinary Malathion level was the variable with the most significant contribution to the risk of AMD compared with other pesticides and demographic variables. Our results show that there are interactive effects between different variables, which jointly affect the occurrence of AMD, and also prove the high reliability and accuracy of the model.

The retina is a fairly complex tissue that plays a crucial role in vision. The retina is divided into the neural epithelium and the pigment epithelium. The neural epithelium contains five main cell types: the cone and rod cells, bipolar cells, amacrine cells, Müller cells, and ganglion cells. The pigment epithelium mainly consists of RPE cells, which are tightly arranged among cells to form the 10-layer structure of the retina. Light signals are converted into electrical and chemical signals through the retina and transmitted to the visual center via the optic nerve to form an image (17).

Because of the complex and delicate structure of the retina, any substance that affects the metabolism of retinal cells will affect the function of the retina. Previous studies have found some chemicals that cause retinal toxicity, such as hydroxychloroquine, which can cause retinal damage (18). Not only drugs, but other substances in nature can also affect the retina and affect the patient’s vision. For example, turmeric (Rosaceae) has great potential for preventing and treating chronic diseases such as arthritis and diabetes (19). Kisu (Rosaceae) is one of the most commonly used medicinal plants in some parts of Africa for treating diarrhea and diabetes (20, 21). In animal experiments, two compounds were given to chicks for feeding, and the results showed that both compounds would damage visual function (visual discrimination and stimulus detection in the peripheral field), and high doses of both drugs could induce neurodegeneration (22).

While pesticides are a type of chemical agent synthesized by human industry for the purpose of killing pests and weeds, excessive intake or chronic exposure can inevitably have an impact on human tissue structure. For example, organophosphate pesticide poisoning, the most common form of pesticide poisoning, can be classified as either acute or chronic poisoning, both of which pose significant threats to human life safety (23). Research has found that the accumulation of a variety of pesticides, including organophosphates, in the body can lead to the development of retinal degeneration, resulting in a decline in retinal cell function and loss of vision. This conclusion has been confirmed by small sample studies and basic research. According to the study of Montgomery et al., AMD was associated with ever use of organochlorine [OR = 2:7 (95% CI, 1:8, 4:0)] and organophosphate [OR = 2:0 (95% CI, 1.3, 3.0)] insecticides and phenoxyacetate herbicides [OR = 1:9 (95% CI, 1:2, 2:8)]. Even when gender is stratified, the results are still significant (8).

Machine learning can efficiently identify the most important factors that affect the outcome, and this process does not require human intervention and continuous improvement (24, 25). Random forests can combine the prediction results of multiple decision trees, providing stronger generalization and stability. Compared with the traditional linear model, it can also deal with the relationship between nonlinear variables. Neural networks also have excellent analytical capabilities in analyzing nonlinear relationships and high-dimensional data, but they usually require a lot of data and computational resources, take a long time to train and are difficult to adjust (26). SVM is not sensitive to data but can handle nonlinear and high-dimensional datasets (27). DT supports visual analysis but is prone to overfitting problems (28). KNN has several beneficial features, including high accuracy, insensitivity to outliers, no assumptions about data input, simplicity and efficiency; however, its time complexity is quite high (29). Besides, Elastic Net, Logistic Regression, LightGBM, and XGBoost show distinct advantages and disadvantages. Logistic Regression, as a classic linear model, offers high interpretability, making it easy to understand variable impacts. However, it struggles with complex data patterns. Elastic Net combines Lasso and Ridge, excelling in handling multicollinearity and variable selection (30). LightGBM and XGBoost, being gradient—boosting algorithms (31, 32), are computationally efficient and highly accurate, but their black—box nature reduces interpretability. We selected the RF model as the best performance model for predicting AMD based on pesticide exposure data using ROC analysis and corresponding AUC values. Random forest (RF) is a popular machine learning algorithm that is particularly good at handling classification and regression problems (33). According to the results of the machine learning modeling analysis, the random forest model has high model predictive value. The area under the ROC curve (AUC) is a widely accepted metric for evaluating binary classification models. An AUC of 0.75 indicates a model that significantly outperforms random chance (AUC = 0.5) and offers practical utility across diverse domains. For example, in clinical diagnostics, Khalilia et al. demonstrated that models with AUC ≥ 0.75 provide “clinically meaningful discrimination” in predicting disease outcomes, even when data are noisy or imbalanced (34). Similarly, in machine learning, Bradley (35) categorized AUC = 0.75 as “moderately accurate,” suitable for applications like fraud detection, where balancing sensitivity and specificity is critical. Additionally, Lobo et al. emphasized that AUC improvements from 0.7 to 0.75 can substantially enhance decision-making in ecology and conservation biology (36). While higher AUC values are ideal, achieving 0.75 reflects a robust compromise between model complexity and real-world applicability, making it a valuable benchmark in contexts demanding actionable yet imperfect predictions. Then, we used SHAP analysis to rank the variables in the random forest model in order of importance, with the top five variables being age, zinc intake, malathion, chlorpyrifos, and BMI. We employed SHAP (SHapley Additive exPlanations) for variable importance analysis due to its “mathematical rigor” and “consistency” in capturing feature contributions within complex models like random forests (37). Unlike Gini importance, which overestimates high-cardinality features, or Permutation Importance, which suffers from collinearity sensitivity and computational cost, SHAP quantifies marginal contributions using Shapley values, ensuring unbiased estimates. The alignment of SHAP-derived rankings with prior studies underscores the robustness of key predictors across methodologies. SHAP additionally enables granular interpretation of feature interactions, enhancing mechanistic insights. It can be seen that age remains the strongest risk factor for AMD, consistent with previous studies. In addition, zinc intake is also important in the development of AMD, with a higher intake of zinc reducing the occurrence of RPE cell autophagy and thus reducing the incidence of AMD (38, 39). In this model, malathion and chlorpyrifos play a more important role in the development of AMD than other pesticides. In the SHAP analysis that only includes pesticide components, Chlorpyrifos, Paranitrophenol, and Malathion remain the top three most important variables.

Chlorpyrifos and Malathion are organophosphate (OP) pesticides. Epidemiological evidence suggests that farmers who use organophosphate pesticides have a higher age-related macular degeneration (AMD) incidence rate (8) This research has also been confirmed in animal models. Both of them affect AChE function and thus have an impact on retinal physiology, as evidenced by the slower recovery of dark-adapted mice in the ERG measurement after intermittent doses of Chlorpyrifos (40). Chlorpyrifos can also promote cell damage through oxidative stress. Oxidative stress and cell death were inhibited in animals pretreated with a combination of antioxidant components such as vitamin C (250 mg/kg) and vitamin E (150 mg/kg) for 6 days. Therefore, oxidative stress promotes organophosphate-induced cell death (41). On the other hand, organophosphate pesticides also inhibit AchE activity and increase intracellular calcium levels, both of which can be blocked by vitamin C and E, further proving that ROS production is the main cause of these effects (42). Chlorpyrifos also causes ROS production in human retinal pigment epithelial cells 19 (ARPE 19 cells) (43). In animal studies, continuous exposure to Chlorpyrifos also reduced anterograde axonal transport from the optic nerve to the superior colliculus in rats (44). It has been shown that organophosphorus pesticides can disrupt the connection between driver proteins and microtubules, which in theory leads to disruption of the driving-dependent vesicle transport in microtubules (45). In summary, prolonged exposure to organophosphorus pesticides reduces the function and activity of optic cells, leading to the progression of AMD.

The strength of this study is the establishment of a predictive model of pesticide exposure and the development of age-related macular degeneration. This model has high predictive value, and the conclusion obtained by using shape analysis to rank the importance of variables is highly consistent with the results of previous studies, so we believe that this model has certain predictive value. However, there are some limitations in this paper. First of all, due to the difference in metabolic rate of pesticides in the body, the duration, dose and frequency of exposure to pesticides may affect the concentration of pesticides in urine. The performance of the random forest model on the verification set needs to be improved, and the prediction performance of the model can be improved by increasing the sample size in the future. In addition, due to the limitation of data collection, only the data from 2007 to 2008 were included, so the conclusions of this study still have certain limitations in generalization and use. Further research is needed to confirm the relationship between the two. Given the inherent limitations of cross-sectional data, we suggest that future studies should rely more on longitudinal data or other experimental designs to further verify whether the associations we found are causal.

## Conclusion

This study used machine learning algorithms to establish a diagnostic model for AMD caused by pesticide exposure, and random forest had the highest predictive value among many models. The importance of variables in the random forest model was ranked, indicating that exposure to malathion and chlorpyrifos is more likely to cause AMD, which suggests that relevant departments should be more cautious when using and producing similar pesticides.

## Data Availability

Publicly available datasets were analyzed in this study. This data can be found at: https://www.cdc.gov/nchs/nhanes/.

## Appendix A List of acronyms.

RIDAGEYR: Age.

RIAGENDR: Gender.

RIDRETH1: Ethnicity.

DMDEDUC2: Level of education.

DMDMARTL: Marital status.

SMQ020: Smoking.

ALQ101: Alcohol use.

BPQ020: Hypertension.

INDFMPIR: Household income.

DR1TLZ: Dietary intake of lutein and zeaxanthin.

DR1TZINC: Dietary intake of zinc.

BMXBMI: BMI.

URX24D: 2,4-dichlorophenoxyacetic acid.

URX4FP: 4-fluoro-3-phenoxybenzoic.

URXCB3: cis-3-(2,2-dibromovinyl)-2,2-dimethylcyclopropane carboxylic acid.

URXCPM: 3,5,6-trichloropyridinol.

URXMAL: Malathion diacid.

URXOPM: 3-phenoxybenzoic.

URXOXY: Oxypyrimidine.

URXPAR: Paranitrophenol.

URXTCC: trans-3-(2,2-dichlorovinyl)-2,2-dimethylcyclopropane carboxylic acid.

URX25T: 2,4,5-Trichlorophenoxyacetic acid.

URXUCR: urine.

## Appendix B Optimal hyperparameters of different models.

**Table:**

dt	cost_complexity tree_depth min_n	0.000237 4 8
rf	mtry trees min_n	10,200 20
xgboost	mtry min_n tree_depth	2 8 3
enet	penalty mixture	0.0774 0.25
svm	cost rbf_sigma	0.0312 0.0001
nent	hidden_units penalty	24 1
lightgbm	mtry trees min_n tree_depth learn_rate loss_reduction	131,494 28 8 0.000000290 0.000302
knn	neighbors	11
