# Supplementary material for: Machine learning model for age related macular degeneration based on pesticides: the National Health and Nutrition Examination Survey 2007–2008
Source: Front Public Health. 2025 Apr 16;13:1561913. doi: 10.3389/fpubh.2025.1561913 (PMC12042703; doi:10.3389/fpubh.2025.1561913)
Supplement: Supplementary file 1 [file Table_1.docx]

library(haven)

library(dplyr)

library(arsenal)

library(survey)

demo <- read_xpt("2007-2008/Demographics/demo_e.xpt")

names(demo)

dput(names(demo))

demo <- demo %>%

select("SEQN","RIAGENDR","RIDAGEYR","RIDRETH1","DMDEDUC2","DMDMARTL","INDFMPIR",

"WTINT2YR", "WTMEC2YR", "SDMVPSU", "SDMVSTRA")

diet <- read_xpt("2007-2008/Dietary/dr1tot_e.xpt")

diet <- diet %>%

select("SEQN","DR1TLZ","DR1TZINC")

bmi <- read_xpt("2007-2008/Examination/bmx_e.xpt")

bmi <- bmi %>%

select("SEQN","BMXBMI","BMXHT","BMXWAIST")

smoke <- read_xpt("2007-2008/Questionnaire/smq_e.xpt")

smoke<- smoke %>%

select("SEQN","SMQ020")

ALQ <- read_xpt("2007-2008/Questionnaire/alq_e.xpt")

ALQ<- ALQ %>%

select("SEQN","ALQ101","ALQ110")

BPQ <- read_xpt("2007-2008/Questionnaire/bpq_e.xpt")

BPQ<- BPQ %>%

select("SEQN","BPQ020")

vision <- read_xpt("2007-2008/Examination/vix_e.xpt")

vision<- vision %>%

select("SEQN","VIXORSM" ,"VIXOLSM")

PES <- read_xpt("2007-2008/Laboratory/uphopm_e.xpt")

PES<- PES %>%

select("SEQN":"URXUCR")

RET <- read_xpt("2007-2008/Examination/opxret_e.xpt")

RET<- RET %>%

select("SEQN","OPXSGLAU","OPXDGLAU","OPDUARM")

GLA <- read_xpt("2007-2008/Questionnaire/viq_e.xpt")

GLA<- GLA %>%

select("SEQN","VIQ090","VIQ310")

data2009 <- full_join(demo,diet,by ="SEQN")

data2009 <- full_join(data2009,bmi,by="SEQN")

data2009 <- full_join(data2009,smoke,by="SEQN")

data2009 <- full_join(data2009,vision,by="SEQN")

data2009 <- full_join(data2009,ALQ,by="SEQN")

data2009 <- full_join(data2009,BPQ,by="SEQN")

data2009 <- full_join(data2009,PES,by="SEQN")

data2009 <- full_join(data2009,RET,by="SEQN")

data2009 <- full_join(data2009,GLA,by="SEQN")

# 模型机器---R语言tidymodels包机器学习分类与回归模型---二分类---SVM

# https://www.tidymodels.org/find/parsnip/

# https://parsnip.tidymodels.org/reference/svm_rbf.html

# https://parsnip.tidymodels.org/reference/details_svm_rbf_kernlab.html

# 模型评估指标

# https://cran.r-project.org/web/packages/yardstick/vignettes/metric-types.html

library(tidymodels)

# 读取数据

Heart <- readr::read_csv(file.choose())

colnames(Heart)

# 修正变量类型

# 将分类变量转换为factor

for(i in c(1,3,4,5,10,11,12,23)){

Heart[[i]] <- factor(Heart[[i]])

}

# 变量类型修正后数据概况

skimr::skim(Heart)

###############################################################

# 数据拆分

set.seed(4321)

datasplit <- initial_split(Heart, prop = 0.75, strata = AMD)

traindata <- training(datasplit)

testdata <- testing(datasplit)

###############################################################

# 数据预处理

# 先对照训练集写配方

datarecipe <- recipe(AMD ~ ., traindata) %>%

step_naomit(all_predictors(), skip = F) %>%

step_dummy(all_nominal_predictors()) %>%

step_center(all_predictors()) %>%

step_scale(all_predictors()) %>%

prep()

datarecipe

# 按方处理训练集和测试集

traindata2 <- bake(datarecipe, new_data = NULL) %>%

dplyr::select(AMD, everything())

testdata2 <- bake(datarecipe, new_data = testdata) %>%

dplyr::select(AMD, everything())

# 数据预处理后数据概况

skimr::skim(traindata2)

skimr::skim(testdata2)

###############################################################

# 训练模型

# 设定模型

model_rsvm <- svm_rbf(

mode = "classification",

engine = "kernlab",

cost = tune(),

rbf_sigma = tune()

)

model_rsvm

# workflow

wk_rsvm <-

workflow() %>%

add_model(model_rsvm) %>%

add_formula(AMD ~ .)

wk_rsvm

# 重抽样设定-5折交叉验证

set.seed(42)

folds <- vfold_cv(traindata2, v = 5)

folds

# 超参数寻优范围

hpset_rsvm <- parameters(cost(range = c(-5, 5)),

rbf_sigma(range = c(-4, -1)))

hpgrid_rsvm <- grid_regular(hpset_rsvm, levels = c(2,3))

hpgrid_rsvm

# 交叉验证网格搜索过程

set.seed(42)

tune_rsvm <- wk_rsvm %>%

tune_grid(resamples = folds,

grid = hpgrid_rsvm,

metrics = metric_set(yardstick::accuracy,

yardstick::roc_auc,

yardstick::pr_auc),

control = control_grid(save_pred = T, verbose = T))

# 图示交叉验证结果

autoplot(tune_rsvm)

eval_tune_rsvm <- tune_rsvm %>%

collect_metrics()

eval_tune_rsvm

# 经过交叉验证得到的最优超参数

hpbest_rsvm <- tune_rsvm %>%

select_best(metric = "roc_auc")

hpbest_rsvm

# 采用最优超参数组合训练最终模型

final_rsvm <- wk_rsvm %>%

finalize_workflow(hpbest_rsvm) %>%

fit(traindata2)

final_rsvm

# 提取最终的算法模型

final_rsvm %>%

extract_fit_engine()

###############################################################

# 应用模型-预测训练集

predtrain_rsvm <- final_rsvm %>%

predict(new_data = traindata2, type = "prob") %>%

bind_cols(traindata2 %>% select(AMD)) %>%

mutate(dataset = "train")

predtrain_rsvm

# 评估模型ROC曲线-训练集上

levels(traindata2$AMD)

roctrain_rsvm <- predtrain_rsvm %>%

roc_curve(AMD, .pred_No, event_level = "first") %>%

mutate(dataset = "train")

roctrain_rsvm

autoplot(roctrain_rsvm)

# 约登法则对应的p值

yueden_rsvm <- roctrain_rsvm %>%

mutate(yueden = sensitivity + specificity - 1) %>%

slice_max(yueden) %>%

pull(.threshold)

yueden_rsvm

# 预测概率+约登法则=预测分类

predtrain_rsvm2 <- predtrain_rsvm %>%

mutate(.pred_class =

factor(ifelse(.pred_No >= yueden_rsvm, "No", "Yes")))

predtrain_rsvm2

# 混淆矩阵

cmtrain_rsvm <- predtrain_rsvm2 %>%

conf_mat(truth = AMD, estimate = .pred_class)

cmtrain_rsvm

autoplot(cmtrain_rsvm, type = "heatmap") +

scale_fill_gradient(low = "white", high = "skyblue") +

theme(text = element_text(size = 15))

# 合并指标

eval_train_rsvm <- cmtrain_rsvm %>%

summary(event_level = "first") %>%

bind_rows(predtrain_rsvm %>%

roc_auc(AMD, .pred_No, event_level = "first")) %>%

mutate(dataset = "train")

eval_train_rsvm

###############################################################

# 应用模型-预测测试集

predtest_rsvm <- final_rsvm %>%

predict(new_data = testdata2, type = "prob") %>%

bind_cols(testdata2 %>% select(AMD)) %>%

mutate(dataset = "test") %>%

mutate(model = "rsvm")

predtest_rsvm

# 评估模型ROC曲线-测试集上

roctest_rsvm <- predtest_rsvm %>%

roc_curve(AMD, .pred_No, event_level = "first") %>%

mutate(dataset = "test")

roctest_rsvm

autoplot(roctest_rsvm)

# 预测概率+约登法则=预测分类

predtest_rsvm2 <- predtest_rsvm %>%

mutate(.pred_class =

factor(ifelse(.pred_No >= yueden_rsvm, "No", "Yes")))

predtest_rsvm2

# 混淆矩阵

cmtest_rsvm <- predtest_rsvm2 %>%

conf_mat(truth = AMD, estimate = .pred_class)

cmtest_rsvm

autoplot(cmtest_rsvm, type = "heatmap") +

scale_fill_gradient(low = "white", high = "skyblue") +

theme(text = element_text(size = 15))

# 合并指标

eval_test_rsvm <- cmtest_rsvm %>%

summary(event_level = "first") %>%

bind_rows(predtest_rsvm %>%

roc_auc(AMD, .pred_No, event_level = "first")) %>%

mutate(dataset = "test")

eval_test_rsvm

###############################################################

# 合并训练集和测试集上ROC曲线

roctrain_rsvm %>%

bind_rows(roctest_rsvm) %>%

mutate(dataset = factor(dataset, levels = c("train", "test"))) %>%

ggplot(aes(x = 1-specificity, y = sensitivity, color = dataset)) +

geom_path(linewidth = 1) +

theme_bw()

# 合并训练集和测试集上性能指标

eval_rsvm <- eval_train_rsvm %>%

bind_rows(eval_test_rsvm) %>%

mutate(model = "rsvm")

eval_rsvm

#############################################################

# 最优超参数的交叉验证指标平均结果

eval_best_cv_rsvm <- eval_tune_rsvm %>%

inner_join(hpbest_rsvm[, 1:2])

eval_best_cv_rsvm

# 最优超参数的交叉验证指标具体结果

eval_best_cv5_rsvm <- tune_rsvm %>%

collect_predictions() %>%

inner_join(hpbest_rsvm[, 1:2]) %>%

group_by(id) %>%

roc_auc(AMD, .pred_No) %>%

ungroup() %>%

mutate(model = "rsvm") %>%

inner_join(eval_best_cv_rsvm[c(3,5,7)])

eval_best_cv5_rsvm

# 保存评估结果

save(final_rsvm,

predtest_rsvm,

eval_rsvm,

eval_best_cv5_rsvm,

file = ".\\cls2\\evalresult_rsvm.RData")

# 最优超参数的交叉验证指标图示

eval_best_cv5_rsvm %>%

filter(.metric == "roc_auc") %>%

ggplot(aes(x = id, y = .estimate, group = 1)) +

geom_point() +

geom_line() +

scale_y_continuous(limits = c(0, 1)) +

labs(x = "", y = "roc_auc") +

theme_bw()

# 最优超参数的交叉验证图示

tune_rsvm %>%

collect_predictions() %>%

inner_join(hpbest_rsvm[, 1:2]) %>%

group_by(id) %>%

roc_curve(AMD, .pred_No) %>%

ungroup() %>%

ggplot(aes(x = 1-specificity, y = sensitivity, color = id)) +

geom_path(linewidth = 1) +

theme_bw()

###################################################################

# 自变量数据集

colnames(traindata2)

traindatax <- traindata2[,-1]

colnames(traindatax)

# iml包

library(iml)

predictor_model <- Predictor$new(

final_rsvm,

data = traindatax,

y = traindata2$AHD,

predict.function = function(model, newdata){

predict(model, newdata, type = "prob") %>%

pull(2)

}

)

# 变量重要性-基于置换

imp_model <- FeatureImp$new(

predictor_model,

loss = function(actual, predicted){

return(1-Metrics::auc(as.numeric(actual=="Yes"), predicted))

}

)

# 数值

imp_model$results

# 图示

imp_model$plot() +

theme_bw()

# 变量效应

pdp_model <- FeatureEffect$new(

predictor_model,

feature = "Age",

method = "pdp"

)

# 数值

pdp_model$results

# 图示

pdp_model$plot() +

theme_bw()

# 所有变量的效应全部输出

effs_model <- FeatureEffects$new(predictor_model, method = "pdp")

# 数值

effs_model$results

# 图示

effs_model$plot()

# 单样本shap分析

shap_model <- Shapley$new(

predictor_model,

x.interest = traindatax[1,]

)

# 数值

shap_model$results

# 图示

shap_model$plot() +

theme_bw()

# 基于所有样本的shap分析

# fastshap包

library(fastshap)

shap <- explain(

final_rsvm,

X = as.data.frame(traindatax),

nsim = 10,

adjust = T,

pred_wrapper = function(model, newdata) {

predict(model, newdata, type = "prob") %>% pull(2)

}

)

# 单样本图示

force_plot(object = shap[1L, ],

feature_values = as.data.frame(traindatax)[1L, ],

baseline = mean(predtrain_rsvm$.pred_Yes),

display = "viewer")

# 变量重要性

autoplot(shap, fill = "skyblue") +

theme_bw()

data1 <- shap %>%

as.data.frame() %>%

dplyr::mutate(id = 1:n()) %>%

pivot_longer(cols = -(ncol(traindatax)+1), values_to = "shap")

shapimp <- data1 %>%

dplyr::group_by(name) %>%

dplyr::summarise(shap.abs.mean = mean(abs(shap))) %>%

dplyr::arrange(shap.abs.mean) %>%

dplyr::mutate(name = forcats::as_factor(name))

data2 <- traindatax %>%

dplyr::mutate(id = 1:n()) %>%

pivot_longer(cols = -(ncol(traindatax)+1))

# 所有变量shap图示

library(ggbeeswarm)

data1 %>%

left_join(data2) %>%

dplyr::rename("feature" = "name") %>%

dplyr::group_by(feature) %>%

dplyr::mutate(

value = (value - min(value)) / (max(value) - min(value)),

feature = factor(feature, levels = levels(shapimp$name))

) %>%

dplyr::arrange(value) %>%

dplyr::ungroup() %>%

ggplot(aes(x = shap, y = feature, color = value)) +

geom_quasirandom(width = 0.2) +

scale_color_gradient(

low = "red",

high = "blue",

breaks = c(0, 1),

labels = c(" Low", "High "),

guide = guide_colorbar(barwidth = 1,

barheight = 20,

ticks = F,

title.position = "right",

title.hjust = 0.5)

) +

labs(x = "SHAP value", color = "Feature value") +

theme_bw() +

theme(legend.title = element_text(angle = -90))

# 单变量shap图示

data1 %>%

left_join(data2) %>%

dplyr::rename("feature" = "name") %>%

dplyr::filter(feature == "MaxHR") %>%

ggplot(aes(x = value, y = shap)) +

geom_point() +

geom_smooth(se = F, span = 0.5) +

labs(x = "MaxHR") +

theme_bw()

#############################################################

# 线性核svm

# https://www.tidymodels.org/find/parsnip/

# https://parsnip.tidymodels.org/reference/svm_linear.html

# https://parsnip.tidymodels.org/reference/details_svm_linear_kernlab.html

model_lsvm <- svm_linear(

mode = "classification",

engine = "kernlab",

cost = tune()

)

hpset_lsvm <- parameters(cost(range = c(-5, 5)))

hpgrid_lsvm <- grid_regular(hpset_lsvm, levels = 5)

hpgrid_lsvm

# 多项式核SVM

# https://www.tidymodels.org/find/parsnip/

# https://parsnip.tidymodels.org/reference/svm_poly.html

# https://parsnip.tidymodels.org/reference/details_svm_poly_kernlab.html

model_psvm <- svm_poly(

mode = "classification",

engine = "kernlab",

cost = tune(),

degree = tune(),

scale_factor = tune()

)

hpset_psvm <- parameters(cost(range = c(-5, 5)),

degree(),

scale_factor(range = c(-5, -1)))

hpgrid_psvm <- grid_regular(hpset_psvm, levels = c(3, 2, 2))

hpgrid_psvm

# 模型机器---R语言tidymodels包机器学习分类与回归模型---二分类---xgboost

# https://www.tidymodels.org/find/parsnip/

# https://parsnip.tidymodels.org/reference/boost_tree.html

# https://parsnip.tidymodels.org/reference/details_boost_tree_xgboost.html

# 模型评估指标

# https://cran.r-project.org/web/packages/yardstick/vignettes/metric-types.html

library(tidymodels)

# 读取数据

Heart <- readr::read_csv(file.choose()) # tibble

colnames(Heart)

# 修正变量类型

# 将分类变量转换为factor

for(i in c(1,3,4,5,10,11,12,23)){

Heart[[i]] <- factor(Heart[[i]])

}

# 变量类型修正后数据概况

skimr::skim(Heart)

###############################################################

# 数据拆分

set.seed(4321)

datasplit <- initial_split(Heart, prop = 0.75, strata = AMD)

traindata <- training(datasplit)

testdata <- testing(datasplit)

###############################################################

# 数据预处理

# 先对照训练集写配方

datarecipe <- recipe(AMD ~ ., traindata) %>%

step_naomit(all_predictors(), skip = F) %>%

step_dummy(all_nominal_predictors()) %>%

prep()

datarecipe

# 按方处理训练集和测试集

traindata2 <- bake(datarecipe, new_data = NULL) %>%

dplyr::select(AMD, everything())

testdata2 <- bake(datarecipe, new_data = testdata) %>%

dplyr::select(AMD, everything())

# 数据预处理后数据概况

skimr::skim(traindata2)

skimr::skim(testdata2)

###############################################################

# 训练模型

# 设定模型

model_xgboost <- boost_tree(

mode = "classification",

engine = "xgboost",

mtry = tune(),

trees = 1000,

min_n = tune(),

tree_depth = tune(),

learn_rate = tune(),

loss_reduction = tune(),

sample_size = tune(),

stop_iter = 25

) %>%

set_args(validation = 0.2)

model_xgboost

# workflow

wk_xgboost <-

workflow() %>%

add_model(model_xgboost) %>%

add_formula(AMD ~ .)

wk_xgboost

# 重抽样设定-5折交叉验证

set.seed(42)

folds <- vfold_cv(traindata2, v = 5)

folds

# 超参数寻优范围

hpset_xgboost <- parameters(

mtry(range = c(2, 8)),

min_n(range = c(5, 20)),

tree_depth(range = c(1, 3)),

learn_rate(range = c(-3, -1)),

loss_reduction(range = c(-3, 0)),

sample_prop(range = c(0.8, 1))

)

# hpgrid_xgboost <-

# grid_regular(hpset_xgboost, levels = c(3, 2, 2, 3, 2, 2))

set.seed(42)

hpgrid_xgboost <- grid_random(hpset_xgboost, size = 5)

hpgrid_xgboost

install.packages("xgboost")

# 交叉验证随机搜索过程

set.seed(42)

tune_xgboost <- wk_xgboost %>%

tune_grid(resamples = folds,

grid = hpgrid_xgboost,

metrics = metric_set(yardstick::accuracy,

yardstick::roc_auc,

yardstick::pr_auc),

control = control_grid(save_pred = T, verbose = T))

# 图示交叉验证结果

autoplot(tune_xgboost)

eval_tune_xgboost <- tune_xgboost %>%

collect_metrics()

eval_tune_xgboost

# 经过交叉验证得到的最优超参数

hpbest_xgboost <- tune_xgboost %>%

select_best(metric = "roc_auc")

hpbest_xgboost

# 采用最优超参数组合训练最终模型

set.seed(42)

final_xgboost <- wk_xgboost %>%

finalize_workflow(hpbest_xgboost) %>%

fit(traindata2)

final_xgboost

# 提取最终的算法模型

final_xgboost2 <- final_xgboost %>%

extract_fit_engine()

# 变量重要性

importance_matrix <- xgb.importance(model = final_xgboost2)

print(importance_matrix)

xgb.plot.importance(importance_matrix = importance_matrix,

measure = "Cover",

col = "skyblue")

# SHAP

colnames(traindata2)

xgb.plot.shap(data = as.matrix(traindata2[,-1]),

model = final_xgboost2,

top_n = 5)

###############################################################

# 应用模型-预测训练集

predtrain_xgboost <- final_xgboost %>%

predict(new_data = traindata2, type = "prob") %>%

bind_cols(traindata2 %>% select(AMD)) %>%

mutate(dataset = "train")

predtrain_xgboost

# 评估模型ROC曲线-训练集上

levels(traindata2$AMD)

roctrain_xgboost <- predtrain_xgboost %>%

roc_curve(AMD, .pred_No, event_level = "first") %>%

mutate(dataset = "train")

roctrain_xgboost

autoplot(roctrain_xgboost)

# 约登法则对应的p值

yueden_xgboost <- roctrain_xgboost %>%

mutate(yueden = sensitivity + specificity - 1) %>%

slice_max(yueden) %>%

pull(.threshold)

yueden_xgboost

# 预测概率+约登法则=预测分类

predtrain_xgboost2 <- predtrain_xgboost %>%

mutate(.pred_class =

factor(ifelse(.pred_No >= yueden_xgboost, "No", "Yes")))

predtrain_xgboost2

# 混淆矩阵

cmtrain_xgboost <- predtrain_xgboost2 %>%

conf_mat(truth = AMD, estimate = .pred_class)

cmtrain_xgboost

autoplot(cmtrain_xgboost, type = "heatmap") +

scale_fill_gradient(low = "white", high = "skyblue") +

theme(text = element_text(size = 15))

# 合并指标

eval_train_xgboost <- cmtrain_xgboost %>%

summary(event_level = "first") %>%

bind_rows(predtrain_xgboost %>%

roc_auc(AMD, .pred_No, event_level = "first")) %>%

mutate(dataset = "train")

eval_train_xgboost

###############################################################

# 应用模型-预测测试集

predtest_xgboost <- final_xgboost %>%

predict(new_data = testdata2, type = "prob") %>%

bind_cols(testdata2 %>% select(AMD)) %>%

mutate(dataset = "test") %>%

mutate(model = "xgboost")

predtest_xgboost

# 评估模型ROC曲线-测试集上

roctest_xgboost <- predtest_xgboost %>%

roc_curve(AMD, .pred_No, event_level = "first") %>%

mutate(dataset = "test")

roctest_xgboost

autoplot(roctest_xgboost)

# 预测概率+约登法则=预测分类

predtest_xgboost2 <- predtest_xgboost %>%

mutate(.pred_class =

factor(ifelse(.pred_No >= yueden_xgboost, "No", "Yes")))

predtest_xgboost2

# 混淆矩阵

cmtest_xgboost <- predtest_xgboost2 %>%

conf_mat(truth = AMD, estimate = .pred_class)

cmtest_xgboost

autoplot(cmtest_xgboost, type = "heatmap") +

scale_fill_gradient(low = "white", high = "skyblue") +

theme(text = element_text(size = 15))

# 合并指标

eval_test_xgboost <- cmtest_xgboost %>%

summary(event_level = "first") %>%

bind_rows(predtest_xgboost %>%

roc_auc(AMD, .pred_No, event_level = "first")) %>%

mutate(dataset = "test")

eval_test_xgboost

###############################################################

# 合并训练集和测试集上ROC曲线

roctrain_xgboost %>%

bind_rows(roctest_xgboost) %>%

mutate(dataset = factor(dataset, levels = c("train", "test"))) %>%

ggplot(aes(x = 1-specificity, y = sensitivity, color = dataset)) +

geom_path(linewidth = 1) +

theme_bw()

# 合并训练集和测试集上性能指标

eval_xgboost <- eval_train_xgboost %>%

bind_rows(eval_test_xgboost) %>%

mutate(model = "xgboost")

eval_xgboost

#############################################################

# 最优超参数的交叉验证指标平均结果

eval_best_cv_xgboost <- eval_tune_xgboost %>%

inner_join(hpbest_xgboost[, 1:6])

eval_best_cv_xgboost

# 最优超参数的交叉验证指标具体结果

eval_best_cv5_xgboost <- tune_xgboost %>%

collect_predictions() %>%

inner_join(hpbest_xgboost[, 1:6]) %>%

group_by(id) %>%

roc_auc(AMD, .pred_No) %>%

ungroup() %>%

mutate(model = "xgboost") %>%

inner_join(eval_best_cv_xgboost[c(7,9,11)])

eval_best_cv5_xgboost

# 保存评估结果

save(final_xgboost,

predtest_xgboost,

eval_xgboost,

eval_best_cv5_xgboost,

file = ".\\cls2\\evalresult_xgboost.RData")

# 最优超参数的交叉验证指标图示

eval_best_cv5_xgboost %>%

filter(.metric == "roc_auc") %>%

ggplot(aes(x = id, y = .estimate, group = 1)) +

geom_point() +

geom_line() +

scale_y_continuous(limits = c(0, 1)) +

labs(x = "", y = "roc_auc") +

theme_bw()

# 最优超参数的交叉验证图示

tune_xgboost %>%

collect_predictions() %>%

inner_join(hpbest_xgboost[, 1:6]) %>%

group_by(id) %>%

roc_curve(AMD, .pred_No, event_level = "first") %>%

ungroup() %>%

ggplot(aes(x = 1-specificity, y = sensitivity, color = id)) +

geom_path(linewidth = 1) +

theme_bw()

###################################################################

# 自变量数据集

colnames(traindata2)

traindatax <- traindata2[,-1]

colnames(traindatax)

# iml包

library(iml)

predictor_model <- Predictor$new(

final_xgboost,

data = traindatax,

y = traindata2$AHD,

predict.function = function(model, newdata){

predict(model, newdata, type = "prob") %>%

pull(2)

}

)

# 变量重要性-基于置换

imp_model <- FeatureImp$new(

predictor_model,

loss = function(actual, predicted){

return(1-Metrics::auc(as.numeric(actual=="Yes"), predicted))

}

)

# 数值

imp_model$results

# 图示

imp_model$plot() +

theme_bw()

# 变量效应

pdp_model <- FeatureEffect$new(

predictor_model,

feature = "Age",

method = "pdp"

)

# 数值

pdp_model$results

# 图示

pdp_model$plot() +

theme_bw()

# 所有变量的效应全部输出

effs_model <- FeatureEffects$new(predictor_model, method = "pdp")

# 数值

effs_model$results

# 图示

effs_model$plot()

# 单样本shap分析

shap_model <- Shapley$new(

predictor_model,

x.interest = traindatax[1,]

)

# 数值

shap_model$results

# 图示

shap_model$plot() +

theme_bw()

# 基于所有样本的shap分析

# fastshap包

library(fastshap)

shap <- explain(

final_xgboost,

X = as.data.frame(traindatax),

nsim = 10,

adjust = T,

pred_wrapper = function(model, newdata) {

predict(model, newdata, type = "prob") %>% pull(2)

}

)

# 单样本图示

force_plot(object = shap[1L, ],

feature_values = as.data.frame(traindatax)[1L, ],

baseline = mean(predtrain_xgboost$.pred_Yes),

display = "viewer")

# 变量重要性

autoplot(shap, fill = "skyblue") +

theme_bw()

data1 <- shap %>%

as.data.frame() %>%

dplyr::mutate(id = 1:n()) %>%

pivot_longer(cols = -(ncol(traindatax)+1), values_to = "shap")

shapimp <- data1 %>%

dplyr::group_by(name) %>%

dplyr::summarise(shap.abs.mean = mean(abs(shap))) %>%

dplyr::arrange(shap.abs.mean) %>%

dplyr::mutate(name = forcats::as_factor(name))

data2 <- traindatax %>%

dplyr::mutate(id = 1:n()) %>%

pivot_longer(cols = -(ncol(traindatax)+1))

# 所有变量shap图示

library(ggbeeswarm)

data1 %>%

left_join(data2) %>%

dplyr::rename("feature" = "name") %>%

dplyr::group_by(feature) %>%

dplyr::mutate(

value = (value - min(value)) / (max(value) - min(value)),

feature = factor(feature, levels = levels(shapimp$name))

) %>%

dplyr::arrange(value) %>%

dplyr::ungroup() %>%

ggplot(aes(x = shap, y = feature, color = value)) +

geom_quasirandom(width = 0.2) +

scale_color_gradient(

low = "red",

high = "blue",

breaks = c(0, 1),

labels = c(" Low", "High "),

guide = guide_colorbar(barwidth = 1,

barheight = 20,

ticks = F,

title.position = "right",

title.hjust = 0.5)

) +

labs(x = "SHAP value", color = "Feature value") +

theme_bw() +

theme(legend.title = element_text(angle = -90))

# 单变量shap图示

data1 %>%

left_join(data2) %>%

dplyr::rename("feature" = "name") %>%

dplyr::filter(feature == "MaxHR") %>%

ggplot(aes(x = value, y = shap)) +

geom_point() +

geom_smooth(se = F, span = 0.5) +

labs(x = "MaxHR") +

theme_bw()

# 模型机器---R语言tidymodels包机器学习分类与回归模型---二分类---决策树

# https://www.tidymodels.org/find/parsnip/

# https://parsnip.tidymodels.org/reference/decision_tree.html

# https://parsnip.tidymodels.org/reference/details_decision_tree_rpart.html

# 模型评估指标

# https://cran.r-project.org/web/packages/yardstick/vignettes/metric-types.html

# install.packages("tidymodels")

library(tidymodels)

# 读取数据

# read.csv()

Heart <- readr::read_csv(file.choose())

colnames(Heart)

# 修正变量类型

# 将分类变量转换为factor

for(i in c(1,3,4,5,10,11,12,23)){

Heart[[i]] <- factor(Heart[[i]])

}

# factor(Heart[,i])

# 变量类型修正后数据概况

skimr::skim(Heart)

##############################################################

# 数据拆分

set.seed(4321)

datasplit <- initial_split(Heart, prop = 0.75, strata = AMD)

traindata <- training(datasplit)

testdata <- testing(datasplit)

##############################################################

# 数据预处理

# 先对照训练集写配方

datarecipe <- recipe(AMD ~ ., traindata) %>%

step_naomit(all_predictors(), skip = F) %>%

step_dummy(all_nominal_predictors()) %>% # 若无需shap图形则可无此步骤

prep()

datarecipe

# 按方处理训练集和测试集

traindata2 <- bake(datarecipe, new_data = NULL) %>%

dplyr::select(AMD, everything())

testdata2 <- bake(datarecipe, new_data = testdata) %>%

dplyr::select(AMD, everything())

# 数据预处理后数据概况

skimr::skim(traindata2)

skimr::skim(testdata2)

##############################################################

# 训练模型

# 设定模型

model_dt <- decision_tree(

mode = "classification",

engine = "rpart",

tree_depth = tune(),

min_n = tune(),

cost_complexity = tune()

) %>%

set_args(model=TRUE)

model_dt

# workflow

wk_dt <-

workflow() %>%

add_model(model_dt) %>%

add_formula(AMD ~ .)

wk_dt

# 重抽样设定-5折交叉验证

set.seed(42)

folds <- vfold_cv(traindata2, v = 5)

folds

# 超参数寻优范围

hpset_dt <- parameters(tree_depth(range = c(3, 7)),

min_n(range = c(5, 10)),

cost_complexity(range = c(-6, -1)))

# hpgrid_dt <- grid_regular(hpset_dt, levels = c(3, 2, 4))

set.seed(42)

hpgrid_dt <- grid_random(hpset_dt, size = 5)

hpgrid_dt

log10(hpgrid_dt$cost_complexity)

# 交叉验证网格搜索过程

set.seed(42)

tune_dt <- wk_dt %>%

tune_grid(resamples = folds,

grid = hpgrid_dt,

metrics = metric_set(yardstick::accuracy,

yardstick::roc_auc,

yardstick::pr_auc),

control = control_grid(save_pred = T, verbose = T))

# 图示交叉验证结果

autoplot(tune_dt)

eval_tune_dt <- tune_dt %>%

collect_metrics()

eval_tune_dt

# 经过交叉验证得到的最优超参数

hpbest_dt <- tune_dt %>%

select_by_one_std_err(metric = "roc_auc", desc(cost_complexity))

hpbest_dt

# 采用最优超参数组合训练最终模型

final_dt <- wk_dt %>%

finalize_workflow(hpbest_dt) %>%

fit(traindata2)

final_dt

# 提取最终的算法模型

final_dt2 <- final_dt %>%

extract_fit_engine()

library(rpart.plot)

rpart.plot(final_dt2)

final_dt2$variable.importance

# par(mar = c(10, 3, 1, 1))

barplot(final_dt2$variable.importance, las = 2)

##############################################################

# 应用模型-预测训练集

predtrain_dt <- final_dt %>%

predict(new_data = traindata2, type = "prob") %>%

bind_cols(traindata2 %>% dplyr::select(AMD)) %>%

mutate(dataset = "train")

predtrain_dt

# 评估模型ROC曲线-训练集上

contrasts(traindata2$AMD)

roctrain_dt <- predtrain_dt %>%

# roc_curve(AHD, .pred_Yes, event_level = "second") %>%

roc_curve(AMD, .pred_No, event_level = "first") %>%

mutate(dataset = "train")

roctrain_dt

autoplot(roctrain_dt)

# 约登法则对应的p值

yueden_dt <- roctrain_dt %>%

mutate(yueden = sensitivity + specificity - 1) %>%

slice_max(yueden) %>%

pull(.threshold)

yueden_dt

# 预测概率+约登法则=预测分类

predtrain_dt2 <- predtrain_dt %>%

mutate(.pred_class =

factor(ifelse(.pred_No >= yueden_dt, "No", "Yes")))

predtrain_dt2

# 混淆矩阵

cmtrain_dt <- predtrain_dt2 %>%

conf_mat(truth = AMD, estimate = .pred_class)

cmtrain_dt

autoplot(cmtrain_dt, type = "heatmap") +

scale_fill_gradient(low = "white", high = "skyblue") +

theme(text = element_text(size = 15))

# 合并指标

eval_train_dt <- cmtrain_dt %>%

summary(event_level = "first") %>%

bind_rows(predtrain_dt %>%

roc_auc(AMD, .pred_No, event_level = "first")) %>%

mutate(dataset = "train")

eval_train_dt

##############################################################

# 应用模型-预测测试集

predtest_dt <- final_dt %>%

predict(new_data = testdata2, type = "prob") %>%

bind_cols(testdata2 %>% dplyr::select(AMD)) %>%

mutate(dataset = "test") %>%

mutate(model = "dt")

predtest_dt

# 评估模型ROC曲线-测试集上

roctest_dt <- predtest_dt %>%

roc_curve(AMD, .pred_No, event_level = "first") %>%

mutate(dataset = "test")

roctest_dt

autoplot(roctest_dt)

# 预测概率+约登法则=预测分类

predtest_dt2 <- predtest_dt %>%

mutate(.pred_class =

factor(ifelse(.pred_No >= yueden_dt, "No", "Yes")))

predtest_dt2

# 混淆矩阵

cmtest_dt <- predtest_dt2 %>%

conf_mat(truth = AMD, estimate = .pred_class)

cmtest_dt

autoplot(cmtest_dt, type = "heatmap") +

scale_fill_gradient(low = "white", high = "skyblue") +

theme(text = element_text(size = 15))

# 合并指标

eval_test_dt <- cmtest_dt %>%

summary(event_level = "first") %>%

bind_rows(predtest_dt %>%

roc_auc(AMD, .pred_No, event_level = "first")) %>%

mutate(dataset = "test")

eval_test_dt

##############################################################

# 合并训练集和测试集上ROC曲线

roctrain_dt %>%

bind_rows(roctest_dt) %>%

mutate(dataset = factor(dataset, levels = c("train", "test"))) %>%

ggplot(aes(x = 1-specificity, y = sensitivity, color = dataset)) +

geom_path(linewidth = 1) +

theme_bw()

# 合并训练集和测试集上性能指标

eval_dt <- eval_train_dt %>%

bind_rows(eval_test_dt) %>%

mutate(model = "dt")

eval_dt

#############################################################

# 最优超参数的交叉验证指标平均结果

eval_best_cv_dt <- eval_tune_dt %>%

inner_join(hpbest_dt[, 1:3])

eval_best_cv_dt

# 最优超参数的交叉验证指标具体结果

eval_best_cv5_dt <- tune_dt %>%

collect_predictions() %>%

inner_join(hpbest_dt[, 1:3]) %>%

group_by(id) %>%

roc_auc(AMD, .pred_No) %>%

ungroup() %>%

mutate(model = "dt") %>%

inner_join(eval_best_cv_dt[c(4,6,8)])

eval_best_cv5_dt

# 保存评估结果

save(final_dt,

predtest_dt,

eval_dt,

eval_best_cv5_dt,

file = ".\\cls2\\evalresult_dt.RData")

# 最优超参数的交叉验证指标图示

eval_best_cv5_dt %>%

filter(.metric == "roc_auc") %>%

ggplot(aes(x = id, y = .estimate, group = 1)) +

geom_point() +

geom_line() +

scale_y_continuous(limits = c(0, 1)) +

labs(x = "", y = "roc_auc") +

theme_bw()

# 最优超参数的交叉验证图示

tune_dt %>%

collect_predictions() %>%

inner_join(hpbest_dt[, 1:3]) %>%

group_by(id) %>%

roc_curve(AMD, .pred_No, event_level = "first") %>%

ungroup() %>%

ggplot(aes(x = 1-specificity, y = sensitivity, color = id)) +

geom_path(linewidth = 1) +

theme_bw()

###################################################################

# 自变量数据集

colnames(traindata2)

traindatax <- traindata2[,-1]

colnames(traindatax)

# iml包

library(iml)

predictor_model <- Predictor$new(

final_dt,

data = traindatax,

y = traindata2$AMD,

predict.function = function(model, newdata){

predict(model, newdata, type = "prob") %>%

pull(2)

}

)

# 变量重要性-基于置换

imp_model <- FeatureImp$new(

predictor_model,

loss = function(actual, predicted){

return(1-Metrics::auc(as.numeric(actual=="Yes"), predicted))

}

)

# 数值

imp_model$results

# 图示

imp_model$plot() +

theme_bw()

# 变量效应

pdp_model <- FeatureEffect$new(

predictor_model,

feature = "RIDAGEYR",

method = "pdp"

)

# 数值

pdp_model$results

# 图示

pdp_model$plot() +

theme_bw()

# 所有变量的效应全部输出

effs_model <- FeatureEffects$new(predictor_model, method = "pdp")

# 数值

effs_model$results

# 图示

effs_model$plot()

# 单样本shap分析

shap_model <- Shapley$new(

predictor_model,

x.interest = traindatax[1,]

)

# 数值

shap_model$results

# 图示

shap_model$plot() +

theme_bw()

# 基于所有样本的shap分析

# fastshap包

library(fastshap)

shap <- explain(

final_dt,

X = as.data.frame(traindatax),

nsim = 10,

adjust = T,

pred_wrapper = function(model, newdata) {

predict(model, newdata, type = "prob") %>% pull(2)

}

)

# 单样本图示

force_plot(object = shap[1L, ],

feature_values = as.data.frame(traindatax)[1L, ],

baseline = mean(predtrain_dt$.pred_Yes),

display = "viewer")

# 变量重要性

autoplot(shap, fill = "skyblue") +

theme_bw()

data1 <- shap %>%

as.data.frame() %>%

dplyr::mutate(id = 1:n()) %>%

pivot_longer(cols = -(ncol(traindatax)+1), values_to = "shap")

shapimp <- data1 %>%

dplyr::group_by(name) %>%

dplyr::summarise(shap.abs.mean = mean(abs(shap))) %>%

dplyr::arrange(shap.abs.mean) %>%

dplyr::mutate(name = forcats::as_factor(name))

data2 <- traindatax %>%

dplyr::mutate(id = 1:n()) %>%

pivot_longer(cols = -(ncol(traindatax)+1))

# 所有变量shap图示

library(ggbeeswarm)

data1 %>%

left_join(data2) %>%

dplyr::rename("feature" = "name") %>%

dplyr::group_by(feature) %>%

dplyr::mutate(

value = (value - min(value)) / (max(value) - min(value)),

feature = factor(feature, levels = levels(shapimp$name))

) %>%

dplyr::arrange(value) %>%

dplyr::ungroup() %>%

ggplot(aes(x = shap, y = feature, color = value)) +

geom_quasirandom(width = 0.2) +

scale_color_gradient(

low = "red",

high = "blue",

breaks = c(0, 1),

labels = c(" Low", "High "),

guide = guide_colorbar(barwidth = 1,

barheight = 20,

ticks = F,

title.position = "right",

title.hjust = 0.5)

) +

labs(x = "SHAP value", color = "Feature value") +

theme_bw() +

theme(legend.title = element_text(angle = -90))

# 单变量shap图示

data1 %>%

left_join(data2) %>%

dplyr::rename("feature" = "name") %>%

dplyr::filter(feature == "MaxHR") %>%

ggplot(aes(x = value, y = shap)) +

geom_point() +

geom_smooth(se = F, span = 0.5) +

labs(x = "MaxHR") +

theme_bw()

# 模型机器---R语言tidymodels包机器学习分类与回归模型---二分类---lasso岭回归弹性网络

# https://www.tidymodels.org/find/parsnip/

# https://parsnip.tidymodels.org/reference/logistic_reg.html

# https://parsnip.tidymodels.org/reference/details_logistic_reg_glmnet.html

# 模型评估指标

# https://cran.r-project.org/web/packages/yardstick/vignettes/metric-types.html

library(tidymodels)

# 读取数据

Heart <- read.csv("AMDNOYES.csv")

Heart <- readr::read_csv(file.choose())

colnames(Heart)

glimpse(Heart)

# 修正变量类型

# 将分类变量转换为factor

for(i in c(1,3,4,5,10,11,12,23)){

Heart[[i]] <- factor(Heart[[i]])

}

# 变量类型修正后数据概况

skimr::skim(Heart)

##############################################################

# 数据拆分

set.seed(4321)

datasplit <- initial_split(Heart, prop = 0.75, strata = AMD)

traindata <- training(datasplit)

testdata <- testing(datasplit)

##############################################################

# 数据预处理

# 先对照训练集写配方

datarecipe <- recipe(AMD ~ ., traindata) %>%

step_naomit(all_predictors(), skip = F) %>%

step_dummy(all_nominal_predictors()) %>%

step_center(all_predictors()) %>%

step_scale(all_predictors()) %>%

prep()

datarecipe

# 按方处理训练集和测试集

traindata2 <- bake(datarecipe, new_data = NULL) %>%

dplyr::select(AMD, everything())

testdata2 <- bake(datarecipe, new_data = testdata) %>%

dplyr::select(AMD, everything())

# 数据预处理后数据概况

skimr::skim(traindata2)

skimr::skim(testdata2)

##############################################################

# 训练模型

# 设定模型

model_enet <- logistic_reg(

mode = "classification",

engine = "glmnet",

# mixture = 1, # LASSO

# mixture = 0, # 岭回归

mixture = tune(),

penalty = tune()

)

model_enet

# workflow

wk_enet <- workflow() %>%

add_model(model_enet) %>%

add_formula(AMD ~ .)

wk_enet

# 设定5折交叉验证

set.seed(42)

folds <- vfold_cv(traindata2, v = 5)

folds

# 超参数寻优范围

hpset_enet <- parameters(mixture(),

penalty(range = c(-5, 0)))

hpgrid_enet <- grid_regular(hpset_enet, levels = c(5, 10))

hpgrid_enet

log10(hpgrid_enet$penalty)

# 交叉验证网格搜索过程

set.seed(42)

tune_enet <- wk_enet %>%

tune_grid(resamples = folds,

grid = hpgrid_enet,

metrics = metric_set(yardstick::accuracy,

yardstick::roc_auc,

yardstick::pr_auc),

control = control_grid(save_pred = T, verbose = T))

# 图示交叉验证结果

autoplot(tune_enet)

eval_tune_enet <- tune_enet %>%

collect_metrics()

eval_tune_enet

# 经过交叉验证得到的最优超参数

hpbest_enet <- tune_enet %>%

select_by_one_std_err(metric = "roc_auc", desc(penalty))

hpbest_enet

# 采用最优超参数组合训练最终模型

set.seed(42)

final_enet <- wk_enet %>%

finalize_workflow(hpbest_enet) %>%

fit(traindata2)

final_enet

# 最终模型对应的系数

final_enet %>% tidy()

# 提取最终的算法模型

final_enet %>%

extract_fit_engine() %>%

plot()

##############################################################

# 应用模型-预测训练集

predtrain_enet <- final_enet %>%

predict(new_data = traindata2, type = "prob") %>%

bind_cols(traindata2 %>% select(AMD)) %>%

mutate(dataset = "train")

predtrain_enet

# 评估模型ROC曲线-训练集上

contrasts(traindata2$AMD)

roctrain_enet <- predtrain_enet %>%

roc_curve(AMD, .pred_No, event_level = "first") %>%

mutate(dataset = "train")

autoplot(roctrain_enet)

# 约登法则对应的p值

yueden_enet <- roctrain_enet %>%

mutate(yueden = sensitivity + specificity - 1) %>%

slice_max(yueden) %>%

pull(.threshold)

yueden_enet

# 预测概率+约登法则=预测分类

predtrain_enet2 <- predtrain_enet %>%

mutate(.pred_class =

factor(ifelse(.pred_No >= yueden_enet, "No", "Yes")))

predtrain_enet2

# 混淆矩阵

cmtrain_enet <- predtrain_enet2 %>%

conf_mat(truth = AMD, estimate = .pred_class)

cmtrain_enet

autoplot(cmtrain_enet, type = "heatmap") +

scale_fill_gradient(low = "white", high = "skyblue") +

theme(text = element_text(size = 15))

# 合并指标

eval_train_enet <- cmtrain_enet %>%

summary(event_level = "first") %>%

bind_rows(predtrain_enet %>%

roc_auc(AMD, .pred_No, event_level = "first")) %>%

mutate(dataset = "train")

eval_train_enet

##############################################################

# 应用模型-预测测试集

predtest_enet <- final_enet %>%

predict(new_data = testdata2, type = "prob") %>%

bind_cols(testdata2 %>% select(AMD)) %>%

mutate(dataset = "test") %>%

mutate(model = "enet")

predtest_enet

# 评估模型ROC曲线-测试集上

roctest_enet <- predtest_enet %>%

roc_curve(AMD, .pred_No, event_level = "first") %>%

mutate(dataset = "test")

autoplot(roctest_enet)

# 预测概率+约登法则=预测分类

predtest_enet2 <- predtest_enet %>%

mutate(.pred_class =

factor(ifelse(.pred_No >= yueden_enet, "No", "Yes")))

predtest_enet2

# 混淆矩阵

cmtest_enet <- predtest_enet2 %>%

conf_mat(truth = AMD, estimate = .pred_class)

cmtest_enet

autoplot(cmtest_enet, type = "heatmap") +

scale_fill_gradient(low = "white", high = "skyblue") +

theme(text = element_text(size = 15))

# 合并指标

eval_test_enet <- cmtest_enet %>%

summary(event_level = "first") %>%

bind_rows(predtest_enet %>%

roc_auc(AMD, .pred_No, event_level = "first")) %>%

mutate(dataset = "test")

eval_test_enet

##############################################################

# 合并训练集和测试集上ROC曲线

roctrain_enet %>%

bind_rows(roctest_enet) %>%

mutate(dataset = factor(dataset, levels = c("train", "test"))) %>%

ggplot(aes(x = 1-specificity, y = sensitivity, color = dataset)) +

geom_path(linewidth = 1) +

theme_bw()

# 合并训练集和测试集上性能指标

eval_enet <- eval_train_enet %>%

bind_rows(eval_test_enet) %>%

mutate(model = "enet")

eval_enet

#############################################################

# 最优超参数的交叉验证指标平均结果

eval_best_cv_enet <- eval_tune_enet %>%

inner_join(hpbest_enet[, 1:2])

eval_best_cv_enet

# 最优超参数的交叉验证指标具体结果

eval_best_cv5_enet <- tune_enet %>%

collect_predictions() %>%

inner_join(hpbest_enet[, 1:2]) %>%

group_by(id) %>%

roc_auc(AMD, .pred_No) %>%

ungroup() %>%

mutate(model = "enet") %>%

inner_join(eval_best_cv_enet[c(3,5,7)])

eval_best_cv5_enet

# 保存评估结果

save(final_enet,

predtest_enet,

eval_enet,

eval_best_cv5_enet,

file = ".\\cls2\\evalresult_enet.RData")

# 最优超参数的交叉验证指标图示

eval_best_cv5_enet %>%

filter(.metric == "roc_auc") %>%

ggplot(aes(x = id, y = .estimate, group = 1)) +

geom_point() +

geom_line() +

scale_y_continuous(limits = c(0, 1)) +

labs(x = "", y = "roc_auc") +

theme_bw()

# 最优超参数的交叉验证图示

tune_enet %>%

collect_predictions() %>%

inner_join(hpbest_enet[, 1:2]) %>%

group_by(id) %>%

roc_curve(AMD, .pred_No) %>%

ungroup() %>%

ggplot(aes(x = 1-specificity, y = sensitivity, color = id)) +

geom_path(linewidth = 1) +

theme_bw()

###################################################################

# 自变量数据集

colnames(traindata2)

traindatax <- traindata2[,-1]

colnames(traindatax)

# iml包

library(iml)

predictor_model <- Predictor$new(

final_enet,

data = traindatax,

y = traindata2$AHD,

predict.function = function(model, newdata){

predict(model, newdata, type = "prob") %>%

pull(2)

}

)

# 变量重要性-基于置换

imp_model <- FeatureImp$new(

predictor_model,

loss = function(actual, predicted){

return(1-Metrics::auc(as.numeric(actual=="Yes"), predicted))

}

)

# 数值

imp_model$results

# 图示

imp_model$plot() +

theme_bw()

# 变量效应

pdp_model <- FeatureEffect$new(

predictor_model,

feature = "Age",

method = "pdp"

)

# 数值

pdp_model$results

# 图示

pdp_model$plot() +

theme_bw()

# 所有变量的效应全部输出

effs_model <- FeatureEffects$new(predictor_model, method = "pdp")

# 数值

effs_model$results

# 图示

effs_model$plot()

# 单样本shap分析

shap_model <- Shapley$new(

predictor_model,

x.interest = traindatax[1,]

)

# 数值

shap_model$results

# 图示

shap_model$plot() +

theme_bw()

# 基于所有样本的shap分析

# fastshap包

library(fastshap)

shap <- explain(

final_enet,

X = as.data.frame(traindatax),

nsim = 10,

adjust = T,

pred_wrapper = function(model, newdata) {

predict(model, newdata, type = "prob") %>% pull(2)

}

)

# 单样本图示

force_plot(object = shap[1L, ],

feature_values = as.data.frame(traindatax)[1L, ],

baseline = mean(predtrain_enet$.pred_Yes),

display = "viewer")

# 变量重要性

autoplot(shap, fill = "skyblue") +

theme_bw()

data1 <- shap %>%

as.data.frame() %>%

dplyr::mutate(id = 1:n()) %>%

pivot_longer(cols = -(ncol(traindatax)+1), values_to = "shap")

shapimp <- data1 %>%

dplyr::group_by(name) %>%

dplyr::summarise(shap.abs.mean = mean(abs(shap))) %>%

dplyr::arrange(shap.abs.mean) %>%

dplyr::mutate(name = forcats::as_factor(name))

data2 <- traindatax %>%

dplyr::mutate(id = 1:n()) %>%

pivot_longer(cols = -(ncol(traindatax)+1))

# 所有变量shap图示

library(ggbeeswarm)

data1 %>%

left_join(data2) %>%

dplyr::rename("feature" = "name") %>%

dplyr::group_by(feature) %>%

dplyr::mutate(

value = (value - min(value)) / (max(value) - min(value)),

feature = factor(feature, levels = levels(shapimp$name))

) %>%

dplyr::arrange(value) %>%

dplyr::ungroup() %>%

ggplot(aes(x = shap, y = feature, color = value)) +

geom_quasirandom(width = 0.2) +

scale_color_gradient(

low = "red",

high = "blue",

breaks = c(0, 1),

labels = c(" Low", "High "),

guide = guide_colorbar(barwidth = 1,

barheight = 20,

ticks = F,

title.position = "right",

title.hjust = 0.5)

) +

labs(x = "SHAP value", color = "Feature value") +

theme_bw() +

theme(legend.title = element_text(angle = -90))

# 单变量shap图示

data1 %>%

left_join(data2) %>%

dplyr::rename("feature" = "name") %>%

dplyr::filter(feature == "MaxHR") %>%

ggplot(aes(x = value, y = shap)) +

geom_point() +

geom_smooth(se = F, span = 0.5) +

labs(x = "MaxHR") +

theme_bw()

# 模型机器---R语言tidymodels包机器学习分类与回归模型---二分类---KNN

# https://www.tidymodels.org/find/parsnip/

# https://parsnip.tidymodels.org/reference/nearest_neighbor.html

# https://parsnip.tidymodels.org/reference/details_nearest_neighbor_kknn.html

# 模型评估指标

# https://cran.r-project.org/web/packages/yardstick/vignettes/metric-types.html

library(tidymodels)

# 读取数据

Heart <- readr::read_csv(file.choose()) # tibble

colnames(Heart)

# 修正变量类型

# 将分类变量转换为factor

for(i in c(1,3,4,5,10,11,12,23)){

Heart[[i]] <- factor(Heart[[i]])

}

# 变量类型修正后数据概况

skimr::skim(Heart)

###############################################################

# 数据拆分

set.seed(4321)

datasplit <- initial_split(Heart, prop = 0.75, strata = AMD)

traindata <- training(datasplit)

testdata <- testing(datasplit)

###############################################################

# 数据预处理

# 先对照训练集写配方

datarecipe <- recipe(AMD ~ ., traindata) %>%

step_naomit(all_predictors(), skip = F) %>%

step_dummy(all_nominal_predictors()) %>%

prep()

datarecipe

# 按方处理训练集和测试集

traindata2 <- bake(datarecipe, new_data = NULL) %>%

dplyr::select(AMD, everything())

testdata2 <- bake(datarecipe, new_data = testdata) %>%

dplyr::select(AMD, everything())

# 数据预处理后数据概况

skimr::skim(traindata2)

skimr::skim(testdata2)

###############################################################

# 训练模型

# 设定模型

model_knn <- nearest_neighbor(

mode = "classification",

engine = "kknn",

neighbors = tune(),

weight_func = "rectangular",

dist_power = 2

)

model_knn

# workflow

wk_knn <-

workflow() %>%

add_model(model_knn) %>%

add_formula(AMD ~ .)

wk_knn

# 重抽样设定-5折交叉验证

set.seed(42)

folds <- vfold_cv(traindata2, v = 5)

folds

# 超参数寻优范围

hpset_knn <- parameters(

neighbors(range = c(3, 11))

)

hpgrid_knn <- grid_regular(hpset_knn, levels = 5)

hpgrid_knn

# 交叉验证随机搜索过程

set.seed(42)

tune_knn <- wk_knn %>%

tune_grid(resamples = folds,

grid = hpgrid_knn,

metrics = metric_set(yardstick::accuracy,

yardstick::roc_auc,

yardstick::pr_auc),

control = control_grid(save_pred = T, verbose = T))

##################################################

# 图示交叉验证结果

autoplot(tune_knn)

eval_tune_knn <- tune_knn %>%

collect_metrics()

eval_tune_knn

# 经过交叉验证得到的最优超参数

hpbest_knn <- tune_knn %>%

select_best(metric = "roc_auc")

hpbest_knn

# 采用最优超参数组合训练最终模型

set.seed(42)

final_knn <- wk_knn %>%

finalize_workflow(hpbest_knn) %>%

fit(traindata2)

final_knn

# 提取最终的算法模型

final_knn2 <- final_knn %>%

extract_fit_engine()

###############################################################

# 应用模型-预测训练集

predtrain_knn <- final_knn %>%

predict(new_data = traindata2, type = "prob") %>%

bind_cols(traindata2 %>% select(AMD)) %>%

mutate(dataset = "train")

predtrain_knn

# 评估模型ROC曲线-训练集上

levels(traindata2$AMD)

roctrain_knn <- predtrain_knn %>%

roc_curve(AMD, .pred_No, event_level = "first") %>%

mutate(dataset = "train")

roctrain_knn

autoplot(roctrain_knn)

# 约登法则对应的p值

yueden_knn <- roctrain_knn %>%

mutate(yueden = sensitivity + specificity - 1) %>%

slice_max(yueden) %>%

pull(.threshold)

yueden_knn

# 预测概率+约登法则=预测分类

predtrain_knn2 <- predtrain_knn %>%

mutate(.pred_class =

factor(ifelse(.pred_No >= yueden_knn, "No", "Yes")))

predtrain_knn2

# 混淆矩阵

cmtrain_knn <- predtrain_knn2 %>%

conf_mat(truth = AMD, estimate = .pred_class)

cmtrain_knn

autoplot(cmtrain_knn, type = "heatmap") +

scale_fill_gradient(low = "white", high = "skyblue") +

theme(text = element_text(size = 15))

# 合并指标

eval_train_knn <- cmtrain_knn %>%

summary(event_level = "first") %>%

bind_rows(predtrain_knn %>%

roc_auc(AMD, .pred_No, event_level = "first")) %>%

mutate(dataset = "train")

eval_train_knn

###############################################################

# 应用模型-预测测试集

predtest_knn <- final_knn %>%

predict(new_data = testdata2, type = "prob") %>%

bind_cols(testdata2 %>% select(AMD)) %>%

mutate(dataset = "test") %>%

mutate(model = "knn")

predtest_knn

# 评估模型ROC曲线-测试集上

roctest_knn <- predtest_knn %>%

roc_curve(AMD, .pred_No, event_level = "first") %>%

mutate(dataset = "test")

roctest_knn

autoplot(roctest_knn)

# 预测概率+约登法则=预测分类

predtest_knn2 <- predtest_knn %>%

mutate(.pred_class =

factor(ifelse(.pred_No >= yueden_knn, "No", "Yes")))

predtest_knn2

# 混淆矩阵

cmtest_knn <- predtest_knn2 %>%

conf_mat(truth = AMD, estimate = .pred_class)

cmtest_knn

autoplot(cmtest_knn, type = "heatmap") +

scale_fill_gradient(low = "white", high = "skyblue") +

theme(text = element_text(size = 15))

# 合并指标

eval_test_knn <- cmtest_knn %>%

summary(event_level = "first") %>%

bind_rows(predtest_knn %>%

roc_auc(AMD, .pred_No, event_level = "first")) %>%

mutate(dataset = "test")

eval_test_knn

###############################################################

# 合并训练集和测试集上ROC曲线

roctrain_knn %>%

bind_rows(roctest_knn) %>%

mutate(dataset = factor(dataset, levels = c("train", "test"))) %>%

ggplot(aes(x = 1-specificity, y = sensitivity, color = dataset)) +

geom_path(linewidth = 1) +

theme_bw()

# 合并训练集和测试集上性能指标

eval_knn <- eval_train_knn %>%

bind_rows(eval_test_knn) %>%

mutate(model = "knn")

eval_knn

#############################################################

# 最优超参数的交叉验证指标平均结果

eval_best_cv_knn <- eval_tune_knn %>%

inner_join(hpbest_knn[, 1])

eval_best_cv_knn

# 最优超参数的交叉验证指标具体结果

eval_best_cv5_knn <- tune_knn %>%

collect_predictions() %>%

inner_join(hpbest_knn[, 1]) %>%

group_by(id) %>%

roc_auc(AMD, .pred_No) %>%

ungroup() %>%

mutate(model = "knn") %>%

inner_join(eval_best_cv_knn[c(2,4,6)])

eval_best_cv5_knn

# 保存评估结果

save(final_knn,

predtest_knn,

eval_knn,

eval_best_cv5_knn,

file = ".\\cls2\\evalresult_knn.RData")

# 最优超参数的交叉验证指标图示

eval_best_cv5_knn %>%

filter(.metric == "roc_auc") %>%

ggplot(aes(x = id, y = .estimate, group = 1)) +

geom_point() +

geom_line() +

scale_y_continuous(limits = c(0, 1)) +

labs(x = "", y = "roc_auc") +

theme_bw()

# 最优超参数的交叉验证图示

tune_knn %>%

collect_predictions() %>%

inner_join(hpbest_knn[, 1]) %>%

group_by(id) %>%

roc_curve(AMD, .pred_No, event_level = "first") %>%

ungroup() %>%

ggplot(aes(x = 1-specificity, y = sensitivity, color = id)) +

geom_path(linewidth = 1) +

theme_bw()

###################################################################

# 自变量数据集

colnames(traindata2)

traindatax <- traindata2[,-1]

colnames(traindatax)

# iml包

library(iml)

predictor_model <- Predictor$new(

final_knn,

data = traindatax,

y = traindata2$AHD,

predict.function = function(model, newdata){

predict(model, newdata, type = "prob") %>%

pull(2)

}

)

# 变量重要性-基于置换

imp_model <- FeatureImp$new(

predictor_model,

loss = function(actual, predicted){

return(1-Metrics::auc(as.numeric(actual=="Yes"), predicted))

}

)

# 数值

imp_model$results

# 图示

imp_model$plot() +

theme_bw()

# 变量效应

pdp_model <- FeatureEffect$new(

predictor_model,

feature = "Age",

method = "pdp"

)

# 数值

pdp_model$results

# 图示

pdp_model$plot() +

theme_bw()

# 所有变量的效应全部输出

effs_model <- FeatureEffects$new(predictor_model, method = "pdp")

# 数值

effs_model$results

# 图示

effs_model$plot()

# 单样本shap分析

shap_model <- Shapley$new(

predictor_model,

x.interest = traindatax[1,]

)

# 数值

shap_model$results

# 图示

shap_model$plot() +

theme_bw()

# 基于所有样本的shap分析

# fastshap包

library(fastshap)

shap <- explain(

final_knn,

X = as.data.frame(traindatax),

nsim = 10,

adjust = T,

pred_wrapper = function(model, newdata) {

predict(model, newdata, type = "prob") %>% pull(2)

}

)

# 单样本图示

force_plot(object = shap[1L, ],

feature_values = as.data.frame(traindatax)[1L, ],

baseline = mean(predtrain_knn$.pred_Yes),

display = "viewer")

# 变量重要性

autoplot(shap, fill = "skyblue") +

theme_bw()

data1 <- shap %>%

as.data.frame() %>%

dplyr::mutate(id = 1:n()) %>%

pivot_longer(cols = -(ncol(traindatax)+1), values_to = "shap")

shapimp <- data1 %>%

dplyr::group_by(name) %>%

dplyr::summarise(shap.abs.mean = mean(abs(shap))) %>%

dplyr::arrange(shap.abs.mean) %>%

dplyr::mutate(name = forcats::as_factor(name))

data2 <- traindatax %>%

dplyr::mutate(id = 1:n()) %>%

pivot_longer(cols = -(ncol(traindatax)+1))

# 所有变量shap图示

library(ggbeeswarm)

data1 %>%

left_join(data2) %>%

dplyr::rename("feature" = "name") %>%

dplyr::group_by(feature) %>%

dplyr::mutate(

value = (value - min(value)) / (max(value) - min(value)),

feature = factor(feature, levels = levels(shapimp$name))

) %>%

dplyr::arrange(value) %>%

dplyr::ungroup() %>%

ggplot(aes(x = shap, y = feature, color = value)) +

geom_quasirandom(width = 0.2) +

scale_color_gradient(

low = "red",

high = "blue",

breaks = c(0, 1),

labels = c(" Low", "High "),

guide = guide_colorbar(barwidth = 1,

barheight = 20,

ticks = F,

title.position = "right",

title.hjust = 0.5)

) +

labs(x = "SHAP value", color = "Feature value") +

theme_bw() +

theme(legend.title = element_text(angle = -90))

# 单变量shap图示

data1 %>%

left_join(data2) %>%

dplyr::rename("feature" = "name") %>%

dplyr::filter(feature == "MaxHR") %>%

ggplot(aes(x = value, y = shap)) +

geom_point() +

geom_smooth(se = F, span = 0.5) +

labs(x = "MaxHR") +

theme_bw()

library(tidymodels)

library(bonsai)

Heart <- readr::read_csv(file.choose()) # tibble

colnames(Heart)

for(i in c(1,3,4,5,10,11,12,23)){

Heart[[i]] <- factor(Heart[[i]])

}

skimr::skim(Heart)

###############################################################

set.seed(4321)

datasplit <- initial_split(Heart, prop = 0.75, strata = AMD)

traindata <- training(datasplit)

testdata <- testing(datasplit)

###############################################################

datarecipe <- recipe(AMD ~ ., traindata) %>%

step_naomit(all_predictors(), skip = F) %>%

step_dummy(all_nominal_predictors()) %>%

prep()

datarecipe

traindata2 <- bake(datarecipe, new_data = NULL) %>%

dplyr::select(AMD, everything())

testdata2 <- bake(datarecipe, new_data = testdata) %>%

dplyr::select(AMD, everything())

skimr::skim(traindata2)

skimr::skim(testdata2)

###############################################################

model_lightgbm <- boost_tree(

mode = "classification",

engine = "lightgbm",

tree_depth = tune(),

trees = tune(),

learn_rate = tune(),

mtry = tune(),

min_n = tune(),

loss_reduction = tune()

)

model_lightgbm

# workflow

wk_lightgbm <-

workflow() %>%

add_model(model_lightgbm) %>%

add_formula(AMD ~ .)

wk_lightgbm

set.seed(42)

folds <- vfold_cv(traindata2, v = 5)

folds

hpset_lightgbm <- parameters(

tree_depth(range = c(1, 3)),

trees(range = c(100, 500)),

learn_rate(range = c(-3, -1)),

mtry(range = c(2, 8)),

min_n(range = c(5, 10)),

loss_reduction(range = c(-3, 0))

)

# hpgrid_lightgbm <- grid_regular(hpset_lightgbm, levels = 2)

set.seed(42)

hpgrid_lightgbm <- grid_random(hpset_lightgbm, size = 5)

hpgrid_lightgbm

set.seed(42)

tune_lightgbm <- wk_lightgbm %>%

tune_grid(resamples = folds,

grid = hpgrid_lightgbm,

metrics = metric_set(yardstick::accuracy,

yardstick::roc_auc,

yardstick::pr_auc),

control = control_grid(save_pred = T, verbose = T))

##################################################

set.seed(42)

tune_lightgbm <- wk_lightgbm %>%

tune_bayes(

resamples = folds,

param_info = extract_parameter_set_dials(wk_lightgbm) %>%

update(mtry = finalize(mtry(), traindata2[,-1])),

initial = 10,

iter = 30,

metrics = metric_set(yardstick::accuracy,

yardstick::roc_auc,

yardstick::pr_auc),

control = control_bayes(save_pred = T, verbose = T, no_improve = 5)

)

##################################################

autoplot(tune_lightgbm)

eval_tune_lightgbm <- tune_lightgbm %>%

collect_metrics()

eval_tune_lightgbm

hpbest_lightgbm <- tune_lightgbm %>%

select_best(metric = "roc_auc")

hpbest_lightgbm

set.seed(42)

final_lightgbm <- wk_lightgbm %>%

finalize_workflow(hpbest_lightgbm) %>%

fit(traindata2)

final_lightgbm

final_lightgbm2 <- final_lightgbm %>%

extract_fit_engine()

lgb.importance(final_lightgbm2, percentage = T)

lgb.plot.importance(lgb.importance(final_lightgbm2, percentage = T))

lgb.interprete(final_lightgbm2,

as.matrix(testdata2[,-1]),

1:2)[[2]]

lgb.plot.interpretation(

lgb.interprete(final_lightgbm2, as.matrix(testdata2[,-1]), 2)[[1]]

)

###############################################################

predtrain_lightgbm <- final_lightgbm %>%

predict(new_data = traindata2, type = "prob") %>%

bind_cols(traindata2 %>% select(AMD)) %>%

mutate(dataset = "train")

predtrain_lightgbm

levels(traindata2$AMD)

roctrain_lightgbm <- predtrain_lightgbm %>%

roc_curve(AMD, .pred_No, event_level = "first") %>%

mutate(dataset = "train")

roctrain_lightgbm

autoplot(roctrain_lightgbm)

yueden_lightgbm <- roctrain_lightgbm %>%

mutate(yueden = sensitivity + specificity - 1) %>%

slice_max(yueden) %>%

pull(.threshold)

yueden_lightgbm

predtrain_lightgbm2 <- predtrain_lightgbm %>%

mutate(.pred_class =

factor(ifelse(.pred_No >= yueden_lightgbm, "No", "Yes")))

predtrain_lightgbm2

cmtrain_lightgbm <- predtrain_lightgbm2 %>%

conf_mat(truth = AMD, estimate = .pred_class)

cmtrain_lightgbm

autoplot(cmtrain_lightgbm, type = "heatmap") +

scale_fill_gradient(low = "white", high = "skyblue") +

theme(text = element_text(size = 15))

eval_train_lightgbm <- cmtrain_lightgbm %>%

summary(event_level = "first") %>%

bind_rows(predtrain_lightgbm %>%

roc_auc(AMD, .pred_No, event_level = "first")) %>%

mutate(dataset = "train")

eval_train_lightgbm

###############################################################

predtest_lightgbm <- final_lightgbm %>%

predict(new_data = testdata2, type = "prob") %>%

bind_cols(testdata2 %>% select(AMD)) %>%

mutate(dataset = "test") %>%

mutate(model = "lightgbm")

predtest_lightgbm

roctest_lightgbm <- predtest_lightgbm %>%

roc_curve(AMD, .pred_No, event_level = "first") %>%

mutate(dataset = "test")

roctest_lightgbm

autoplot(roctest_lightgbm)

predtest_lightgbm2 <- predtest_lightgbm %>%

mutate(.pred_class =

factor(ifelse(.pred_No >= yueden_lightgbm, "No", "Yes")))

predtest_lightgbm2

cmtest_lightgbm <- predtest_lightgbm2 %>%

conf_mat(truth = AMD, estimate = .pred_class)

cmtest_lightgbm

autoplot(cmtest_lightgbm, type = "heatmap") +

scale_fill_gradient(low = "white", high = "skyblue") +

theme(text = element_text(size = 15))

eval_test_lightgbm <- cmtest_lightgbm %>%

summary(event_level = "first") %>%

bind_rows(predtest_lightgbm %>%

roc_auc(AMD, .pred_No, event_level = "first")) %>%

mutate(dataset = "test")

eval_test_lightgbm

###############################################################

roctrain_lightgbm %>%

bind_rows(roctest_lightgbm) %>%

mutate(dataset = factor(dataset, levels = c("train", "test"))) %>%

ggplot(aes(x = 1-specificity, y = sensitivity, color = dataset)) +

geom_path(linewidth = 1) +

theme_bw()

eval_lightgbm <- eval_train_lightgbm %>%

bind_rows(eval_test_lightgbm) %>%

mutate(model = "lightgbm")

eval_lightgbm

#############################################################

eval_best_cv_lightgbm <- eval_tune_lightgbm %>%

inner_join(hpbest_lightgbm[, 1:6])

eval_best_cv_lightgbm

eval_best_cv5_lightgbm <- tune_lightgbm %>%

collect_predictions() %>%

inner_join(hpbest_lightgbm[, 1:6]) %>%

group_by(id) %>%

roc_auc(AMD, .pred_No) %>%

ungroup() %>%

mutate(model = "lightgbm") %>%

inner_join(eval_best_cv_lightgbm[c(7,9,11)])

eval_best_cv5_lightgbm

save(final_lightgbm,

predtest_lightgbm,

eval_lightgbm,

eval_best_cv5_lightgbm,

file = ".\\cls2\\evalresult_lightgbm.RData")

model_file <-

tempfile(pattern = "lightgbm", tmpdir = ".", fileext = ".txt")

lightgbm::lgb.save(final_lightgbm2, model_file)

eval_best_cv5_lightgbm %>%

filter(.metric == "roc_auc") %>%

ggplot(aes(x = id, y = .estimate, group = 1)) +

geom_point() +

geom_line() +

scale_y_continuous(limits = c(0, 1)) +

labs(x = "", y = "roc_auc") +

theme_bw()

tune_lightgbm %>%

collect_predictions() %>%

inner_join(hpbest_lightgbm[, 1:6]) %>%

group_by(id) %>%

roc_curve(AMD, .pred_No, event_level = "first") %>%

ungroup() %>%

ggplot(aes(x = 1-specificity, y = sensitivity, color = id)) +

geom_path(linewidth = 1) +

theme_bw()

# 模型机器---R语言tidymodels包机器学习分类与回归模型---二分类---logistic回归

# https://www.tidymodels.org/find/parsnip/

# https://parsnip.tidymodels.org/reference/logistic_reg.html

# https://parsnip.tidymodels.org/reference/details_logistic_reg_glm.html

# 模型评估指标

# https://cran.r-project.org/web/packages/yardstick/vignettes/metric-types.html

library(tidymodels)

# 读取数据

Heart <- readr::read_csv(file.choose()) # tibble

colnames(Heart)

# 修正变量类型

# 将分类变量转换为factor

for(i in c(1,3,4,5,10,11,12,23)){

Heart[[i]] <- factor(Heart[[i]])

}

# 变量类型修正后数据概况

skimr::skim(Heart)

##############################################################

# 数据拆分

set.seed(4321)

datasplit <- initial_split(Heart, prop = 0.75, strata = AMD)

traindata <- training(datasplit)

testdata <- testing(datasplit)

##############################################################

# 数据预处理

# 先对照训练集写配方

datarecipe <- recipe(AMD ~ ., traindata) %>%

step_naomit(all_predictors(), skip = F) %>%

step_dummy(all_nominal_predictors()) %>%

prep()

datarecipe

# 按方处理训练集和测试集

traindata2 <- bake(datarecipe, new_data = NULL) %>%

dplyr::select(AMD, everything())

testdata2 <- bake(datarecipe, new_data = testdata) %>%

dplyr::select(AMD, everything())

# 数据预处理后数据概况

skimr::skim(traindata2)

skimr::skim(testdata2)

##############################################################

# 训练模型

# 设定模型

model_logistic <- logistic_reg(

mode = "classification",

engine = "glm"

)

model_logistic

# 拟合模型

fit_logistic <- model_logistic %>%

fit(AMD ~ ., traindata2)

fit_logistic

fit_logistic$fit

summary(fit_logistic$fit)

# 系数输出

fit_logistic %>%

tidy()

##############################################################

# 应用模型-预测训练集

predtrain_logistic <- fit_logistic %>%

predict(new_data = traindata2, type = "prob") %>%

bind_cols(traindata2 %>% select(AMD)) %>%

mutate(dataset = "train")

predtrain_logistic

# 评估模型ROC曲线-训练集上

levels(traindata2$AMD)

roctrain_logistic <- predtrain_logistic %>%

roc_curve(AMD, .pred_No, event_level = "first") %>%

mutate(dataset = "train")

roctrain_logistic

autoplot(roctrain_logistic)

# 约登法则对应的p值

yueden_logistic <- roctrain_logistic %>%

mutate(yueden = sensitivity + specificity - 1) %>%

slice_max(yueden) %>%

pull(.threshold)

yueden_logistic

# 预测概率+约登法则=预测分类

predtrain_logistic2 <- predtrain_logistic %>%

mutate(.pred_class =

factor(ifelse(.pred_No >= yueden_logistic, "No", "Yes")))

predtrain_logistic2

# 混淆矩阵

cmtrain_logistic <- predtrain_logistic2 %>%

conf_mat(truth = AMD, estimate = .pred_class)

cmtrain_logistic

autoplot(cmtrain_logistic, type = "heatmap") +

scale_fill_gradient(low = "white", high = "skyblue") +

theme(text = element_text(size = 15))

# 合并指标

eval_train_logistic <- cmtrain_logistic %>%

summary(event_level = "first") %>%

bind_rows(predtrain_logistic %>%

roc_auc(AMD, .pred_No, event_level = "first")) %>%

mutate(dataset = "train")

eval_train_logistic

##############################################################

# 应用模型-预测测试集

predtest_logistic <- fit_logistic %>%

predict(new_data = testdata2, type = "prob") %>%

bind_cols(testdata2 %>% select(AMD)) %>%

mutate(dataset = "test") %>%

mutate(model = "logistic")

predtest_logistic

# 评估模型ROC曲线-测试集上

roctest_logistic <- predtest_logistic %>%

roc_curve(AMD, .pred_No, event_level = "first") %>%

mutate(dataset = "test")

autoplot(roctest_logistic)

# 预测概率+约登法则=预测分类

predtest_logistic2 <- predtest_logistic %>%

mutate(.pred_class =

factor(ifelse(.pred_No >= yueden_logistic, "No", "Yes")))

predtest_logistic2

# 混淆矩阵

cmtest_logistic <- predtest_logistic2 %>%

conf_mat(truth = AMD, estimate = .pred_class)

cmtest_logistic

autoplot(cmtest_logistic, type = "heatmap") +

scale_fill_gradient(low = "white", high = "skyblue") +

theme(text = element_text(size = 15))

# 合并指标

eval_test_logistic <- cmtest_logistic %>%

summary(event_level = "first") %>%

bind_rows(predtest_logistic %>%

roc_auc(AMD, .pred_No, event_level = "first")) %>%

mutate(dataset = "test")

eval_test_logistic

##############################################################

# 合并训练集和测试集上ROC曲线

roctrain_logistic %>%

bind_rows(roctest_logistic) %>%

mutate(dataset = factor(dataset, levels = c("train", "test"))) %>%

ggplot(aes(x = 1-specificity, y = sensitivity, color = dataset)) +

geom_path(linewidth = 1) +

theme_bw()

# 合并训练集和测试集上性能指标

eval_logistic <- eval_train_logistic %>%

bind_rows(eval_test_logistic) %>%

mutate(model = "logistic")

eval_logistic

#################################################################

#################################################################

# 设定5折交叉验证

set.seed(42)

folds <- vfold_cv(traindata2, v = 5)

folds

# workflow

wf_logistic <-

workflow() %>%

add_model(model_logistic) %>%

add_formula(AMD ~ .)

wf_logistic

# 交叉验证

set.seed(42)

cv_logistic <-

wf_logistic %>%

fit_resamples(folds,

metrics = metric_set(yardstick::accuracy,

yardstick::roc_auc,

yardstick::pr_auc),

control = control_resamples(save_pred = T))

cv_logistic

# 交叉验证指标平均结果

eval_cv_logistic <- collect_metrics(cv_logistic)

eval_cv_logistic

# 交叉验证指标具体结果

eval_cv5_logistic <- collect_predictions(cv_logistic) %>%

group_by(id) %>%

roc_auc(AMD, .pred_No, event_level = "first") %>%

ungroup() %>%

mutate(model = "logistic") %>%

left_join(eval_cv_logistic[c(1,3,5)])

eval_cv5_logistic

# 保存评估结果

save(fit_logistic,

predtest_logistic,

eval_logistic,

eval_cv5_logistic,

file = ".\\cls2\\evalresult_logistic.RData")

# 交叉验证指标图示

eval_cv5_logistic %>%

filter(.metric == "roc_auc") %>%

ggplot(aes(x = id, y = .estimate, group = 1)) +

geom_point() +

geom_line() +

scale_y_continuous(limits = c(0, 1)) +

labs(x = "", y = "roc_auc") +

theme_bw()

# 交叉验证图示

collect_predictions(cv_logistic) %>%

group_by(id) %>%

roc_curve(AMD, .pred_No, event_level = "first") %>%

ungroup() %>%

ggplot(aes(x = 1-specificity, y = sensitivity, color = id)) +

geom_path(linewidth = 1) +

theme_bw()

###################################################################

# 自变量数据集

colnames(traindata2)

traindatax <- traindata2[,-1]

colnames(traindatax)

# iml包

library(iml)

predictor_model <- Predictor$new(

fit_logistic,

data = traindatax,

y = traindata2$AHD,

predict.function = function(model, newdata){

predict(model, newdata, type = "prob") %>%

pull(2)

}

)

# 变量重要性-基于置换

imp_model <- FeatureImp$new(

predictor_model,

loss = function(actual, predicted){

return(1-Metrics::auc(as.numeric(actual=="Yes"), predicted))

}

)

# 数值

imp_model$results

# 图示

imp_model$plot() +

theme_bw()

# 变量效应

pdp_model <- FeatureEffect$new(

predictor_model,

feature = "Age",

method = "pdp"

)

# 数值

pdp_model$results

# 图示

pdp_model$plot() +

theme_bw()

# 所有变量的效应全部输出

effs_model <- FeatureEffects$new(predictor_model, method = "pdp")

# 数值

effs_model$results

# 图示

effs_model$plot()

# 单样本shap分析

shap_model <- Shapley$new(

predictor_model,

x.interest = traindatax[1,]

)

# 数值

shap_model$results

# 图示

shap_model$plot() +

theme_bw()

# 基于所有样本的shap分析

# fastshap包

library(fastshap)

shap <- explain(

fit_logistic,

X = as.data.frame(traindatax),

nsim = 10,

adjust = T,

pred_wrapper = function(model, newdata) {

predict(model, newdata, type = "prob") %>% pull(2)

}

)

# 单样本图示

force_plot(object = shap[1L, ],

feature_values = as.data.frame(traindatax)[1L, ],

baseline = mean(predtrain_logistic$.pred_Yes),

display = "viewer")

# 变量重要性

autoplot(shap, fill = "skyblue") +

theme_bw()

data1 <- shap %>%

as.data.frame() %>%

dplyr::mutate(id = 1:n()) %>%

pivot_longer(cols = -(ncol(traindatax)+1), values_to = "shap")

shapimp <- data1 %>%

dplyr::group_by(name) %>%

dplyr::summarise(shap.abs.mean = mean(abs(shap))) %>%

dplyr::arrange(shap.abs.mean) %>%

dplyr::mutate(name = forcats::as_factor(name))

data2 <- traindatax %>%

dplyr::mutate(id = 1:n()) %>%

pivot_longer(cols = -(ncol(traindatax)+1))

# 所有变量shap图示

library(ggbeeswarm)

data1 %>%

left_join(data2) %>%

dplyr::rename("feature" = "name") %>%

dplyr::group_by(feature) %>%

dplyr::mutate(

value = (value - min(value)) / (max(value) - min(value)),

feature = factor(feature, levels = levels(shapimp$name))

) %>%

dplyr::arrange(value) %>%

dplyr::ungroup() %>%

ggplot(aes(x = shap, y = feature, color = value)) +

geom_quasirandom(width = 0.2) +

scale_color_gradient(

low = "red",

high = "blue",

breaks = c(0, 1),

labels = c(" Low", "High "),

guide = guide_colorbar(barwidth = 1,

barheight = 20,

ticks = F,

title.position = "right",

title.hjust = 0.5)

) +

labs(x = "SHAP value", color = "Feature value") +

theme_bw() +

theme(legend.title = element_text(angle = -90))

# 单变量shap图示

data1 %>%

left_join(data2) %>%

dplyr::rename("feature" = "name") %>%

dplyr::filter(feature == "MaxHR") %>%

ggplot(aes(x = value, y = shap)) +

geom_point() +

geom_smooth(se = F, span = 0.5) +

labs(x = "MaxHR") +

theme_bw()

# 模型机器---R语言tidymodels包机器学习分类与回归模型---二分类---单隐藏层神经网络

# https://www.tidymodels.org/find/parsnip/

# https://parsnip.tidymodels.org/reference/mlp.html

# https://parsnip.tidymodels.org/reference/details_mlp_nnet.html

# 模型评估指标

# https://cran.r-project.org/web/packages/yardstick/vignettes/metric-types.html

library(tidymodels)

# 读取数据

Heart <- readr::read_csv(file.choose())

colnames(Heart)

# 修正变量类型

# 将分类变量转换为factor

for(i in c(1,3,4,5,10,11,12,23)){

Heart[[i]] <- factor(Heart[[i]])

}

# factor(Heart[,4])

# 变量类型修正后数据概况

skimr::skim(Heart)

#############################################################

# 数据拆分

set.seed(4321)

datasplit <- initial_split(Heart, prop = 0.75, strata = AMD)

traindata <- training(datasplit)

testdata <- testing(datasplit)

#############################################################

# 数据预处理

# 先对照训练集写配方

# recipes包

datarecipe <- recipe(AMD ~ ., traindata) %>%

step_naomit(all_predictors(), skip = F) %>%

step_dummy(all_nominal_predictors()) %>%

step_range(all_predictors()) %>%

prep()

datarecipe

# 按方处理训练集和测试集

traindata2 <- bake(datarecipe, new_data = NULL) %>%

dplyr::select(AMD, everything())

testdata2 <- bake(datarecipe, new_data = testdata) %>%

dplyr::select(AMD, everything())

# 数据预处理后数据概况

skimr::skim(traindata2)

skimr::skim(testdata2)

#############################################################

# 训练模型

# 设定模型

model_mlp <- mlp(

mode = "classification",

engine = "nnet",

hidden_units = tune(),

penalty = tune(),

epochs = tune()

) %>%

set_args(MaxNWts = 5000)

model_mlp

# workflow

wk_mlp <-

workflow() %>%

add_model(model_mlp) %>%

add_formula(AMD ~ .)

wk_mlp

# 重抽样设定-5折交叉验证

set.seed(42)

folds <- vfold_cv(traindata2, v = 5)

folds

# 超参数寻优范围

hpset_mlp <- parameters(hidden_units(range = c(15, 24)),

penalty(range = c(-3, 0)),

epochs(range = c(50, 150)))

hpgrid_mlp <- grid_regular(hpset_mlp, levels = 2)

hpgrid_mlp

# 交叉验证网格搜索过程

set.seed(42)

tune_mlp <- wk_mlp %>%

tune_grid(resamples = folds,

grid = hpgrid_mlp,

metrics = metric_set(yardstick::accuracy,

yardstick::roc_auc,

yardstick::pr_auc),

control = control_grid(save_pred = T, verbose = T))

# 图示交叉验证结果

autoplot(tune_mlp)

eval_tune_mlp <- tune_mlp %>%

collect_metrics()

eval_tune_mlp

# 经过交叉验证得到的最优超参数

hpbest_mlp <- tune_mlp %>%

select_best(metric = "roc_auc")

hpbest_mlp

# 采用最优超参数组合训练最终模型

set.seed(42)

final_mlp <- wk_mlp %>%

finalize_workflow(hpbest_mlp) %>%

fit(traindata2)

final_mlp

# 提取最终的算法模型

final_mlp2 <- final_mlp %>%

extract_fit_engine()

library(NeuralNetTools)

plotnet(final_mlp2)

garson(final_mlp2) +

coord_flip()

olden(final_mlp2) +

coord_flip()

#############################################################

# 应用模型-预测训练集

predtrain_mlp <- final_mlp %>%

predict(new_data = traindata2, type = "prob") %>%

bind_cols(traindata2 %>% select(AMD)) %>%

mutate(dataset = "train")

predtrain_mlp

# 评估模型ROC曲线-训练集上

levels(traindata2$AMD)

roctrain_mlp <- predtrain_mlp %>%

roc_curve(AMD, .pred_No, event_level = "first") %>%

mutate(dataset = "train")

roctrain_mlp

autoplot(roctrain_mlp)

# 约登法则对应的p值

yueden_mlp <- roctrain_mlp %>%

mutate(yueden = sensitivity + specificity - 1) %>%

slice_max(yueden) %>%

pull(.threshold)

yueden_mlp

# 预测概率+约登法则=预测分类

predtrain_mlp2 <- predtrain_mlp %>%

mutate(.pred_class =

factor(ifelse(.pred_No >= yueden_mlp, "No", "Yes")))

predtrain_mlp2

# 混淆矩阵

cmtrain_mlp <- predtrain_mlp2 %>%

conf_mat(truth = AMD, estimate = .pred_class)

cmtrain_mlp

autoplot(cmtrain_mlp, type = "heatmap") +

scale_fill_gradient(low = "white", high = "skyblue") +

theme(text = element_text(size = 15))

# 合并指标

eval_train_mlp <- cmtrain_mlp %>%

summary(event_level = "first") %>%

bind_rows(predtrain_mlp %>%

roc_auc(AMD, .pred_No, event_level = "first")) %>%

mutate(dataset = "train")

eval_train_mlp

#############################################################

# 应用模型-预测测试集

predtest_mlp <- final_mlp %>%

predict(new_data = testdata2, type = "prob") %>%

bind_cols(testdata2 %>% select(AMD)) %>%

mutate(dataset = "test") %>%

mutate(model = "mlp")

predtest_mlp

# 评估模型ROC曲线-测试集上

roctest_mlp <- predtest_mlp %>%

roc_curve(AMD, .pred_No, event_level = "first") %>%

mutate(dataset = "test")

roctest_mlp

autoplot(roctest_mlp)

# 预测概率+约登法则=预测分类

predtest_mlp2 <- predtest_mlp %>%

mutate(.pred_class =

factor(ifelse(.pred_No >= yueden_mlp, "No", "Yes")))

predtest_mlp2

# 混淆矩阵

cmtest_mlp <- predtest_mlp2 %>%

conf_mat(truth = AMD, estimate = .pred_class)

cmtest_mlp

autoplot(cmtest_mlp, type = "heatmap") +

scale_fill_gradient(low = "white", high = "skyblue") +

theme(text = element_text(size = 15))

# 合并指标

eval_test_mlp <- cmtest_mlp %>%

summary(event_level = "first") %>%

bind_rows(predtest_mlp %>%

roc_auc(AMD, .pred_No, event_level = "first")) %>%

mutate(dataset = "test")

eval_test_mlp

#############################################################

# 合并训练集和测试集上ROC曲线

roctrain_mlp %>%

bind_rows(roctest_mlp) %>%

mutate(dataset = factor(dataset, levels = c("train", "test"))) %>%

ggplot(aes(x = 1-specificity, y = sensitivity, color = dataset)) +

geom_path(linewidth = 1) +

theme_bw()

# 合并训练集和测试集上性能指标

eval_mlp <- eval_train_mlp %>%

bind_rows(eval_test_mlp) %>%

mutate(model = "mlp")

eval_mlp

#############################################################

# 最优超参数的交叉验证指标平均结果

eval_best_cv_mlp <- eval_tune_mlp %>%

inner_join(hpbest_mlp[, 1:3])

eval_best_cv_mlp

# 最优超参数的交叉验证指标具体结果

eval_best_cv5_mlp <- tune_mlp %>%

collect_predictions() %>%

inner_join(hpbest_mlp[, 1:3]) %>%

group_by(id) %>%

roc_auc(AMD, .pred_No) %>%

ungroup() %>%

mutate(model = "mlp") %>%

inner_join(eval_best_cv_mlp[c(4,6,8)])

eval_best_cv5_mlp

# 保存评估结果

save(final_mlp,

predtest_mlp,

eval_mlp,

eval_best_cv5_mlp,

file = ".\\cls2\\evalresult_mlp.RData")

# 最优超参数的交叉验证指标图示

eval_best_cv5_mlp %>%

filter(.metric == "roc_auc") %>%

ggplot(aes(x = id, y = .estimate, group = 1)) +

geom_point() +

geom_line() +

scale_y_continuous(limits = c(0, 1)) +

labs(x = "", y = "roc_auc") +

theme_bw()

# 最优超参数的交叉验证图示

tune_mlp %>%

collect_predictions() %>%

inner_join(hpbest_mlp[, 1:3]) %>%

group_by(id) %>%

roc_curve(AMD, .pred_No) %>%

ungroup() %>%

ggplot(aes(x = 1-specificity, y = sensitivity, color = id)) +

geom_path(linewidth = 1) +

theme_bw()

# 模型机器---R语言tidymodels包机器学习分类与回归模型---二分类---模型比较

library(tidymodels)

# 加载各个模型的评估结果

evalfiles <- list.files(".\\cls2\\", full.names = T)

lapply(evalfiles, load, .GlobalEnv)

#############################################################

# 各个模型在测试集上的误差指标

eval <- bind_rows(

eval_logistic, eval_enet, eval_dt,

eval_rf, eval_xgboost, eval_rsvm, eval_mlp,

eval_lightgbm, eval_knn

)

eval

# 平行线图

eval %>%

filter(dataset == "test") %>%

ggplot(aes(x = .metric, y = .estimate, color = model)) +

geom_point() +

geom_line(aes(group = model)) +

theme_bw() +

theme(axis.text.x = element_text(angle = 30, hjust = 1))

# 各个模型在测试集上的误差指标表格

eval2 <- eval %>%

select(-.estimator) %>%

filter(dataset == "test") %>%

pivot_wider(names_from = .metric, values_from = .estimate)

eval2

# 各个模型在测试集上的误差指标图示

eval2 %>%

ggplot(aes(x = model, y = roc_auc, fill = model)) +

geom_col(width = 0.3, show.legend = F) +

geom_text(aes(label = round(roc_auc, 2)),

nudge_y = -0.03) +

theme_bw()
